# Mitigation of TDP-43 toxic phenotype by an RGNEF fragment in amyotrophic lateral sclerosis models

**DOI:** 10.1093/brain/awae078

**Published:** 2024-05-13

**Authors:** Cristian A Droppelmann, Danae Campos-Melo, Veronica Noches, Crystal McLellan, Robert Szabla, Taylor A Lyons, Hind Amzil, Benjamin Withers, Brianna Kaplanis, Kirti S Sonkar, Anne Simon, Emanuele Buratti, Murray Junop, Jamie M Kramer, Michael J Strong

**Affiliations:** Molecular Medicine Group, Robarts Research Institute, Schulich School of Medicine and Dentistry, Western University, London, Ontario N6A 5C1, Canada; Molecular Medicine Group, Robarts Research Institute, Schulich School of Medicine and Dentistry, Western University, London, Ontario N6A 5C1, Canada; Molecular Medicine Group, Robarts Research Institute, Schulich School of Medicine and Dentistry, Western University, London, Ontario N6A 5C1, Canada; Molecular Medicine Group, Robarts Research Institute, Schulich School of Medicine and Dentistry, Western University, London, Ontario N6A 5C1, Canada; Department of Biochemistry, Schulich School of Medicine and Dentistry, Western University, London, Ontario N6A 5C1, Canada; Molecular Medicine Group, Robarts Research Institute, Schulich School of Medicine and Dentistry, Western University, London, Ontario N6A 5C1, Canada; Molecular Medicine Group, Robarts Research Institute, Schulich School of Medicine and Dentistry, Western University, London, Ontario N6A 5C1, Canada; Molecular Medicine Group, Robarts Research Institute, Schulich School of Medicine and Dentistry, Western University, London, Ontario N6A 5C1, Canada; Department of Biochemistry, Schulich School of Medicine and Dentistry, Western University, London, Ontario N6A 5C1, Canada; International Centre for Genetic Engineering and Biotechnology (ICGEB), AREA Science Park, 34149 Trieste, Italy; Department of Biology, Faculty of Science, Western University, London, Ontario N6A 5B7, Canada; International Centre for Genetic Engineering and Biotechnology (ICGEB), AREA Science Park, 34149 Trieste, Italy; Department of Biochemistry, Schulich School of Medicine and Dentistry, Western University, London, Ontario N6A 5C1, Canada; Department of Biochemistry and Molecular Biology, Faculty of Medicine, Dalhousie University, Halifax, Nova Scotia B3H 4R2, Canada; Molecular Medicine Group, Robarts Research Institute, Schulich School of Medicine and Dentistry, Western University, London, Ontario N6A 5C1, Canada; Department of Clinical Neurological Sciences, Schulich School of Medicine and Dentistry, Western University, London, Ontario N6A 5C1, Canada

**Keywords:** amyotrophic lateral sclerosis, motor neuron disease, RNA binding proteins, RNA metabolism, neuronal cytoplasmic inclusions, therapeutic

## Abstract

Aggregation of the RNA-binding protein TAR DNA binding protein (TDP-43) is a hallmark of TDP-proteinopathies including amyotrophic lateral sclerosis (ALS) and frontotemporal dementia (FTD). As TDP-43 aggregation and dysregulation are causative of neuronal death, there is a special interest in targeting this protein as a therapeutic approach. Previously, we found that TDP-43 extensively co-aggregated with the dual function protein GEF (guanine exchange factor) and RNA-binding protein rho guanine nucleotide exchange factor (RGNEF) in ALS patients. Here, we show that an N-terminal fragment of RGNEF (NF242) interacts directly with the RNA recognition motifs of TDP-43 competing with RNA and that the IPT/TIG domain of NF242 is essential for this interaction.

Genetic expression of NF242 in a fruit fly ALS model overexpressing TDP-43 suppressed the neuropathological phenotype increasing lifespan, abolishing motor defects and preventing neurodegeneration. Intracerebroventricular injections of AAV9/NF242 in a severe TDP-43 murine model (rNLS8) improved lifespan and motor phenotype, and decreased neuroinflammation markers.

Our results demonstrate an innovative way to target TDP-43 proteinopathies using a protein fragment with a strong affinity for TDP-43 aggregates and a mechanism that includes competition with RNA sequestration, suggesting a promising therapeutic strategy for TDP-43 proteinopathies such as ALS and FTD.

## Teaser

Droppelmann *et al.* show that an N-terminal fragment of a protein called RGNEF (NF242) competes with RNA to interact directly with TDP-43. In fruit fly and mouse models of ALS, NF242 extended lifespan and reduced motor impairments, suggesting that this approach could be a promising therapeutic strategy for TDP-43 proteinopathies.

## Introduction

Amyotrophic lateral sclerosis (ALS), also known as Lou Gehrig’s disease, is a neurodegenerative disorder characterized by progressive loss of voluntary muscle function, typically leading to death from respiratory failure within 3 to 5 years of symptom onset.^1^ To date, despite multiple efforts, there is no effective therapy that arrests the progression of ALS because of the complex nature of its pathology.^2^ The hallmark of ALS is the presence of unique protein inclusions, the most common of which are composed of RNA-binding proteins (RBPs)^3^ such as TAR DNA binding protein (TDP-43), fused in sarcoma/translocated in liposarcoma (FUS/TLS), TATA-Box binding protein associated factor 15 (TAF 15), Ewing sarcoma breakpoint region 1 (EWS), RNA binding motif protein 45 (RBM45), heterogeneous nuclear ribonucleoprotein A1 and A2/B1 (hnRNPA1 and hnRNPA2B1) and rho guanine nucleotide exchange factor (RGNEF/p190RhoGEF).^4-13^ Of these proteins, TDP-43 is the most extensively studied ALS-associated RBP as its dysregulation has been directly associated with neuronal death *in vitro* and *in vivo*^14,15^ and TDP-43 immunoreactive neuronal cytoplasmic inclusions (NCIs) are observed in 97% of ALS cases.^16^ TDP-43 is also the main pathological component of a group of diseases called TDP-43 proteinopathies, which include frontotemporal dementia (FTD) and limbic-predominant age-related TDP-43 encephalopathy (LATE).^17^ Because of this, there is a special interest in targeting TDP-43 as a therapeutic approach.^18-20^

Previously, we described that RGNEF forms extensive NCIs that co-aggregate with TDP-43 in motor neurons of ALS patients^11,12^ and observed that RGNEF works as a survival factor under stress conditions *in vitro.*^21^ Also, we described that the N-terminal fragment of RGNEF, called NF242 (NH_2_-terminal fragment of 242 amino acids) in this study, is part of a high molecular weight complex with TDP-43 *in vitro* and that both co-localize under metabolic stress conditions.^22^

Here, we hypothesized that NF242 works as a modifier of TDP-43 toxicity *in vivo*. To test this, we studied (i) the mechanism of protein-protein interaction between NF242 and TDP-43 *in vitro* and *in silico*; (ii) the co-expression of RGNEF or NF242 with TDP-43 in *Drosophila melanogaster*; and (iii) the viral ectopic expression of NF242 in an aggressive murine model of ALS (rNLS8).^23^

## Material and methods

### Antibodies, chemicals and plasmids

Antibodies and other critical material used in this study are listed in Supplementary Table 1.

### Constructs

To develop the transgenic flies, the coding regions of TDP-43^wt^, RGNEF and flag-NF242 (previously described^22^) were cloned in the pTW-UASt vector (Drosophila Genomics Resource Center), generating the pTW-TDP-43^wt^, pTW-RGNEF and pTW-flag-NF242 vectors. For the luciferase reporter assay, the coding region of TDP-43^wt^ was cloned into the pcDNA-myc-His-A vector, generating the pcDNA-TDP-43-myc plasmid. For the complementation reporter assay (NanoBiT), the coding region of TDP-43^wt^, TDP-43-ΔNLS (nuclear localization signal of TDP-43 from amino acids 78 to 84 eliminated by site-directed mutagenesis), RGNEF and NF242 were cloned into the pBiT1.1-C [TK LgBiT], pBiT1.1-N [TK LgBiT], pBiT2.1-C [TK SmBiT] and pBiT2.1-N [TK SmBiT] (Promega) vectors. pBiT constructs are detailed in Supplementary Table 2. For surface plasmon resonance spectroscopy (SPR) experiments the pQE30-TDP-43-RRM1 and pQE30-TDP-43-RRM2 plasmids used to express His-RRM-1 (amino acids 101 to 191 of TDP-43) and His-RRM-2 (amino acids 177 to 262 of TDP-43) were generated. The expression plasmid pQE30-TDP-43^1–269^ was used to express His-TDP-43^1–269^ (amino acids 1 to 261 of TDP-43, which include the N-terminal region and both RNA recognition motifs (RRMs). The expression plasmid pBAD-HisA-GST-TDP-43Cri was used to express His-GST-TDP-43^wt^. The expression plasmid pDEST566-RGNEF-275 was used to express His-MBP-RGNEF^1–275^.

### Cell lines

HEK293T cells (ATCC) were maintained in 25 mM glucose, 1 mM pyruvate Dulbecco’s modified Eagle medium (Gibco, Life Technologies) containing 100 U/ml penicillin, 100 U/ml streptomycin (Gibco, Life Technologies), 5 µg/ml plasmocin (InvivoGen) and 10% fetal bovine serum (Gibco, Life Technologies).

### Flies

Stocks and crosses of *Drosophila melanogaster* were cultured according to standard procedures and on standard fly food (water, yeast, cornmeal, brown sugar, agar, propionic acid, 10% methylparaben) (Bloomington Drosophila Stock Center). Flies were raised on 25°C and 70% humidity at a 12-h day/night cycle.

*UAS-TDP-43^wt^*, *UAS-RGNEF* and *UAS-flag-NF242* transgenic lines were generated by random germline insertion into *w1118* flies (*w^−^*) (BestGene). *GMR-Gal4*, *D42-Gal4* and *elav-Gal4* driver lines were obtained from the Bloomington Drosophila Stock Center (Indiana University, Bloomington, Indiana, USA). The flies from stock centres used in this study are listed in Supplementary Table 3.

Single transgenic flies homozygous for the transgene were used in the generation of the double transgenic fly lines, as well as in crosses with Gal4 drivers. Genotypes of the transgenic flies used in this study are listed in Supplementary Table 4.

### Mice lines

Mice strains B6C3F1/J (JAX: 100010), B6; C3-Tg(NEFH-tTA)8Vle/J (JAX:025397) and B6; C3-Tg(tetO-TARDBP*)4Vle/J (JAX:014650) were purchased from The Jackson Laboratory. Experimental double transgenic mouse B6; C3-Tg(NEFH-tTA)8Vle Tg(tetO-TARDBP* (rNLS8) was generated after crossing JAX:025397 and JAX:014650. Double transgenic animals and the breeding pairs were maintained with doxycycline (Dox, 50 μg/ml) in the drinking water to suppress the expression of TDP-43.^23^ The wild-type (wt) control mice for the experiments were obtained from the progeny of the crosses between JAX:025397 and JAX:014650 that were negative for both transgenes. For the experiments, rNLS8 males were excluded due to the observation of a urinary retention problem previously described for this transgenic line.^24^

### Study approval and animal housing

All procedures involving animals, surgeries and animal maintenance were in accordance with the Canadian Council for Animal Care and the University Council on Animal Care guidelines for research. Ethics review and approval was granted by the Animal Care Committee of The University of Western Ontario (Protocol #2020-004). Mice were housed in the ACVS (Animal Care and Veterinary Services) in a temperature-controlled room (21–23°C) with a 12-h light-dark cycle. Animals were given free access to standard rodent chow and were provided with moistened chow on the cage floor and purified dietary supplement (Clear H2O DietGel 76A), after the Dox was removed from the drinking water.

### Transfections

Cell transfection of the constructs was performed using lipofectamine 2000 (Invitrogen) for the cytotoxicity assays or Magnetofection™ (OZ Biosciences) for the complementation reporter assay according to the manufacturer’s protocol.

### Complementation reporter assay: NanoBiT

Protein-protein interaction was analysed using the NanoBit Protein:Protein Interaction (PPI) System (Promega) according to the manufacturer’s instructions. Briefly, cells were seeded in white 96-well plates at 10 000 cells/ml per well and 24 h after were transfected with the pBiT constructs listed in Supplementary Table 1. After 48 h, luciferase activity was measured using the Nano-Glo Live Cell Assay System (Promega) using a Luminometer (Modulus; Turner Biosystems). The expression of the constructs was evaluated by semi-quantitative PCR using 18S as reference gene. There was no statistical difference between the expression of all the NanoBiT constructs used (Supplementary Fig. 1).

### Protein purification

His-GST-TDP-43^wt^, His-TDP-43^1–269^, His-RRM-1, His-RRM-2, His-TDP-43^101–261^ and His-MBP-RGNEF^1–275^ recombinant proteins were purified from *Escherichia coli* using the nickel-immobilized metal affinity chromatography (Ni-IMAC) method. For a detailed protocol, refer to the Supplementary material, ‘Methods’ section.

Purified His-TDP-43^1–102^ was generously provided by Dr Stanley Dunn from the Department of Biochemistry at Western University (London, Canada). His-TDP-43^1–102^ contains an N-terminal 6xHistidine-Thioredoxin tag followed by the first 102 amino acids of human TDP-43.

### SDS-PAGE and western blot

To evaluate the purity of the purified proteins, SDS-PAGE and western blot were performed (Supplementary Fig. 2). The protein aliquots from each purification were run in 4–20% Mini-PROTEAN® TGX™ Precast gradient gels. Gels were stained with Imperial™ Protein Stain (Thermo Scientific) or transferred to a nitrocellulose membrane. For the western blot the membrane was blocked in 5% bovine serum albumin (BSA) made in 1× TBST and the primary antibody (rabbit TDP-43) was incubated at 4°C with shaking overnight followed by horseradish peroxide (HRP) conjugated secondary antibodies for 60 min, at room temperature. Immunoblots were visualized using Western Lightning Plus Chemiluminescence Substrate (Perkin Elmer).

### Surface plasmon resonance spectroscopy

Protein interactions were assessed using a Reichert 2SPR, SR7500DC System. Standard amine coupling (EDC/NHS chemistry) was used to capture purified His-MBP-RGNEF^1–275^ on a carboxymethyl dextran hydrogel sensor chip (Reichert). The amount of ligand immobilized ranged from 2000 to 8000 µRIU. TDP-43 analyte proteins were serially diluted to the concentrations indicated in running buffer. His-GST-TDP-43^wt^ analysis was carried out using running buffer containing 20 mM HEPES pH 7.5, 50 mM KCl, 0.5 mM MgCl_2_, 50 mM NaCl and 0.05% Tween-20. His-TDP-43^1–269^, His-RRM1, His-RRM-2 or His-TDP-43^1–102^ analysis was carried out in running buffer of 1× PBS and 0.1% Tween-20. In the experiments, 50–100 μl of TDP-43 analyte concentrations were injected on both the ligand and reference channels at 5–20 μl/min for 4–7 min with a 1–8-min dissociation time at 22°C. His-MBP protein (Supplementary Table 1) was used as control to evaluate possible unspecific binding. For the competition experiment, a running buffer containing 10 mM Tris pH 8.0, 150 mM NaCl, 2 mM MgCl_2_, 0.05% Tween-20, 1 μM BSA was used. The RNA oligo 5′-GUGUGUGAAUGAAUAAA-3′^25^ biotinylated at 3′ was bound to a neutravidin planar mSAM chip (neutravidin covalently immobilized on a planar mSAM surface, Reichert). The amount of RNA immobilized was 80 RIU. His-TDP-43^1–269^ and His-MBP-RGNEF^1–275^ were pre-incubated in running buffer 30 min at room temperature before injection. Buffer and His-MBP-RGNEF^1–275^ injections to the RNA chip were used as blanks for the experiments. Sensorgrams analysis and dissociation constant (K_D_) calculations were performed using Reichert SPRAutolink (version 1.1.16), TraceDrawer (version 1.8.1) and GraphPad Prism 9.5 software packages.

### TDP-43-RGNEF interaction modelling

#### RGNEF domain analysis

The atomic coordinates of RGNEF residues 1–242 were extracted from the AlphaFold Protein structure database^26^ under the accession Q8N1W1. This model was queried for structural similarity against the entire PDB databank using the DALI protein structure comparison server.^27^ The DALI results were analysed using DALIview (https://github.com/rszabla/daliview) to reveal structurally similar domain families.

#### Structural prediction of the TDP-43/RGNEF heterodimer

Constrained and unconstrained molecular docking of RGNEF^1–242^ onto TDP-43^96–269^ were performed using InterEvDock3 and ClusPro, respectively.^28,29^ The atomic coordinates of RGNEF^1–242^ were taken from the AlphaFold structure database while those of TDP-43 were taken from the available nuclear magnetic resonance (NMR) structure.^25^ To generate the dimer model, the sequence of RGNEF^1–242^ (Uniprot accession Q8N1W1) and the sequence of full-length TDP-43 (Uniprot Q13148) were both used as inputs for AlphaFold2, running in complex prediction mode on ColabFold.^30,31^ The top-scoring output model was used for further structural minimizations. For this, the structure of TDP-43 in the dimer structure was limited to the two RRM domains with about eight additional flanking residues on either side (residues 96–269).^32^ The top-scoring model was used for residue-contact analysis.

The relative structural stability of the dimer was quantified by measuring the conformational spread between all output models. This was done using a custom PyMOL script that aligned each of the 1000 output models against the top-scoring model for the dimer and calculating a root-mean-square deviation (RMSD) value for each model. The top 50 scoring models for the dimer were deposited to the ModelArchive database as a multi-model PDB file. For TDP-43^96–269^ + RGNEF^1–242^ → Accession#: ma-hepyb; password: ZcgpOeyLZf.

TDP-43^(96–269)^ bound to a 12-mer strand of RNA was also minimized from experimental NMR coordinates^25^ (PDB accession: 4BS2)

### *NEFL* mRNA stabilization activity

*NEFL* 3′ untranslated region (UTR) stability by TDP-43 (pCDNA-TDP-43-myc) was studied using a luciferase reporter assay, as previously described,^12^ with minor modifications (pcDNA-flag-NF242 plasmid^22^ was used for the co-expression of flag-NF242).

### Cytotoxicity analysis

Cells were seeded in white 96-well plates at 9000 cells/ml per well. Cytotoxicity was measured using the CytoTox-Glo™ Cytotoxicity Assay kit (Promega) according to the manufacturer’s protocol after 2 days of transfection. To obtain the percentage of cell toxicity, the values obtained after the stress condition or control were normalized against total protease activity obtained after cell lysis using digitonin.

### Expression analysis in flies

To check the expression of the TDP-43, RGNEF, NF242 or green fluorescent protein (GFP) in our fly models, total RNA from at least 15 flies was isolated using TRIzol™ reagent (Invitrogen). Reverse transcription was performed using the SuperScript II reverse transcriptase system (Invitrogen). PCR reactions (Supplementary Fig. 3) were performed using primers listed in Supplementary Table 5.

### Lifespan analysis in flies

F1 male progeny of transgenic flies *elav*>*RGNEF*, *elav*>*RGNEF;TDP-43*, *elav*>*NF242;TDP-43*, *elav*>*GFP;TDP-43*, *D42*>*RGNEF;TDP-43*, *D42*>*NF242;TDP-43* and *D42*>*GFP;TDP-43* were collected and maintained in vials in an incubator set to 25°C at 70% humidity with controlled day/night cycles. The number of dead and live flies were counted every other day. Heterozygote driver lines *elav*>*w^−^*, *D42*>*w^−^*, and non-expressing *UAS-RGNEF* flies were used as additional controls.

### Motor analysis in flies

F1 male progeny of transgenic flies *elav*>*RGNEF*, *elav*>*RGNEF;TDP-43*, *elav*>*NF242;TDP-43*, *elav*>*GFP;TDP-43*, *D42*>*RGNEF;TDP-43*, *D42*>*NF242;TDP-43* and *D42*>*GFP;TDP-43* were collected to evaluate the negative geotaxis (locomotion) using a climbing assay. To achieve this, flies were transferred to a graduated cylinder (24 cm height, 3 cm diameter) divided into four vertical quadrants (from the lower part: Quadrants 1 to 3 = 5 cm each, Quadrant 4 = 9 cm) and sealed with parafilm. Flies were tapped to the bottom of the cylinder and the number of flies present in each quadrant was recorded at 10 and 20 s. Measurements were repeated a total of four times every 3 days. Climbing index was calculated using the formula:


(1) Climbingscore=[Q1+(Q2×2)+(Q3×3)+(Q4×4)]/Totalnumberofflies

where Q represents the number of flies in the respective quadrant.^33^

### Fly eye degeneration

F1 male progeny of transgenic flies *GMR*>*RGNEF*, *GMR*>*NF242*, *GMR*>*RGNEF;TDP-43*, *GMR*>*NF242;TDP-43, GMR*>*GFP;TDP-43*, *GMR*>*C936R* and *GMR*>*w^−^* were collected after 5 days of age to capture images of fly eyes. Flies were anaesthetized with CO_2_ and then photographed using a Leica S9i Stereomicroscope (Leica Microsystems Inc.).

### Fixation of fly tissues

The fixation protocol from the Shcherbata group^34^ was performed to obtain paraffin-embedded adult flies. Whole flies in collars were first incubated in Carnoy’s solution containing absolute ethanol, chloroform and glacial acetic acid 6:3:1 ratio, overnight at 4°C. Flies were then dehydrated by incubation in 40% ethanol for 20 min, 70% ethanol for 20 min and twice in 100% ethanol for 10 min each. Afterwards, flies were incubated in methylbenzoate and methylbenzoate with paraffin solution, 1:1 ratio, for 30 min each at 60°C, following which they were incubated twice in paraffin solution for 60 min each at 60°C. Flies in paraffin were then allowed to solidify at room temperature overnight before cutting the paraffin-embedded flies into 7 μm sections (Pathology Core Facility, Robarts Research Institute). Haematoxylin-eosin staining of selected slides was performed for checking quality and anatomy visualization.

### Immunofluorescence for flies and mice

For slide deparaffinizing, sections of fly brain and eye tissue, mice brain, or mice spinal cord were first seated on a slide warmer at 60°C for 30 min. Slides were then rehydrated in a series of graded alcohols and water. Antigen retrieval was performed in a pressure cooker for 30 min at 100°C in a buffer containing 10 mM citric acid, 2 mM EDTA and 0.05% Tween-20 pH 6.2 for fly tissues or in 10 mM sodium citrate, 0.05% Tween, pH 6.0 for mouse tissues. Next, slides were incubated for 60 min at room temperature in PBS pH 7.2 blocking solution with 5% BSA and 0.3% Triton-X 100, and with primary antibodies at 4°C overnight in a humidifying chamber. After the washes, slides were incubated with Alexa Fluor secondary antibodies for 60 min at room temperature. Dilutions for primary and secondary antibodies are indicated in Supplementary Table 6. For nuclear staining, slides were then incubated with 2 μg/ml Hoechst for 3 min. Alternatively, for flies, anti-histone H3 antibody and SPY555-DNA were used for nuclear staining. After the washes and once dry, coverslips were mounted to the slides using a fluorescent mounting media (Dako). Slides were examined using an SP8 lightening confocal microscopy system (Leica Microsystems Inc.). For the super-resolution stimulated emission depletion (STED) microscopy images, a Leica STELLARIS STED microscope (Leica Microsystems Inc.) was used. The multi-STED method was performed using Alexa-488 and Alexa-595 fluorophores and the 592 and 775 nm STED depletion lasers for tau-STED analysis. All images were visualized using the LAS X 2.0 software (Leica Microsystems Inc.).

### Co-localization images

Intensity correlation analysis^35^ using ImageJ software was performed to obtain the co-localization images. Co-localized pixels are shown as PDM (product of the differences from the mean) images. PDM = (red intensity − mean red intensity) × (green intensity − mean green intensity). In the co-localization images, blue and purple colours indicate lower level of co-localization while yellow and white indicate a high level of co-localization.

### Intracerebroventricular injections of adeno-associated viruses

Self-complementary adeno-associated viruses (AAVs) serotype 9 for neuronal-specific expression of GFP (AAV9/GFP) and NF242 (AAV9/NF242) were produced to a yield of 2.0 × 10^13^ GC/ml and 2.4 × 10^13^ GC/ml, respectively (Vector Biolabs) using pscAAV-GFP and pscAAV-NF242 plasmids.^22^ Mice were stereotaxically injected with the AAVs intraventricularly in the brain (injection site: AP = −0.4 mm; ML = −1.0, +1.0 mm; and DV = 2.3 mm from Bregma) with 2.5 μl (bilateral) of AAV9/GFP or AAV9/NF242 at a rate of 1 μl/min with a 33-G Hamilton syringe. One week after the surgery Dox was removed from the double transgenic mice to induce the expression of TDP-43-ΔNLS.

### Motor analysis in mice

Motor tests for the mice were performed once per week from Dox retrieval.

#### Clasping

Mice were suspended by the tail ∼30 cm above the cage and slowly lowered. Clasping of both hindlimbs that was maintained for ∼30 s was recorded as a positive response.^36^

#### Grip-strength assessment

Front limbs strength was assessed using a Model Grip Strength meter (Columbus Instruments) horizontally mounted. Mice had to grip a wire bail attached to a force transducer sensing shaft (Chatillon 2LBF AMETEK). The peak force of five trials was the grip strength expressed in normalized force (N/g).^37,38^

#### Rotarod

To test motor coordination and balance,^37^ mice were placed on a rotarod apparatus (AccuRotor Rota-Rod, Omnithech electronics, Inc.; software, Fusion 6.4 AccuRotor edition) at a speed of 4 rpm with increased linearly acceleration up to 40 rpm over 300 s. After the initial training session, weekly session of four trials were performed for each animal and the average in the latency to fall of four trials was calculated.

#### Catwalk

CatWalk XT® Version 10.6 system by Noldus was used for mice gait assessment. Tests were conducted in a room with red light and the analysis was made with the average of two videos per animal. Runs were analysed using Noldus software.^39,40^

#### Open field

Mice were placed in a square arena (20 cm × 20 cm) (AccuScan Instruments Inc.) and activity for 20 min was recorded by infra-red photo beams along the *x*-, *y*-, *z*-axes using software Fusion V5 VersaMax Edition. Distance travelled (converted from beam breaks to cm) was recorded at 5-min blocks.^41^ The results of open field were not compared with their wild-type counterpart because of the hypermobility associated with TDP-43 transgenic models,^42,43^ which creates a different basal for transgenic mice when compared with wild-type mice.

### Mice end point

Disease end stage in mice was defined as: CS 4 (clinical score; functional paralysis of both hindlimbs), CS 4+ (CS 4 plus loss of body weight ≥ 20% or body condition score <2) and CS 5 (CS 4 plus righting reflex >20 s).^44^

### Pathology quantification

Relative fluorescence intensity of TDP-43, GFAP and Iba1 staining in the ventral horn of the lumbar spinal cord or brain cortex of rNLS8 mice injected with AAV9/GFP and AAV9/NF242 was measured using LAS X 2.0 software (Leica), quantifying the intensity on at least five different slices (technical replicates) for each animal.

### Statistical analysis

The statistical analyses were performed with GraphPad Prism 9.5 software. Log-rank (Mantel-Cox) test analysis was used to compare lifespan curves and clasping. For the protein-protein experiments one-way ANOVA with Dunnett’s *post hoc* or Student’s *t*-test were performed. For the NanoBiT constructs expression analysis one-way ANOVA with Tukey’s *post hoc* was performed. For the animal motor test studies two-way ANOVA analysis comparing the difference between treatments was performed. For the pathology quantification Student’s *t*-test were performed. Data were expressed as mean ± standard error of the mean (SEM). Data were judged to be statistically significant when *P* < 0.05.

## Results

### Interaction between NF242 and TDP-43

Previously, we observed that RGNEF and TDP-43 co-localize and co-immunoprecipitate and that NF242, an N-terminal fragment of RGNEF encompassing its first 242 amino acids, and TDP-43 are part of a high molecular complex.^12,22^ To evaluate if the interaction between RGNEF with TDP-43 is direct, we performed a complementation reporter assay (NanoBiT)^45^ in HEK293T cells. We transfected a series of constructs containing RGNEF, NF242, TDP-43^ΔNLS^ and TDP-43^wt^ fused to the large or small subunit of the luciferase (Supplementary Fig. 4A and B). TDP-43^ΔNLS^ has previously been described to emulate pathological conditions and localizes in the cytoplasm,^46^ which we thought would facilitate the interaction with RGNEF (mainly cytoplasmic^12,22^). The amino- or C-terminal end of TDP-43^ΔNLS^ fused to the large subunit of luciferase showed interaction with both RGNEF and NF242, but only when the C-terminal end of the latter proteins was fused to the small subunit of the luciferase (Supplementary Figs 4C and 5A–D). In experiments with TDP-43^wt^, we observed interaction only with NF242 and when both proteins had the luciferase subunit fused to the C-terminal end (Supplementary Figs 4C and 5E–H). We previously observed that NF242 localizes both in the nucleus and the cytoplasm in cultured cells.^22^ This facilitates its interaction with TDP-43^wt^ and aligns with our results.

**Figure 1:**
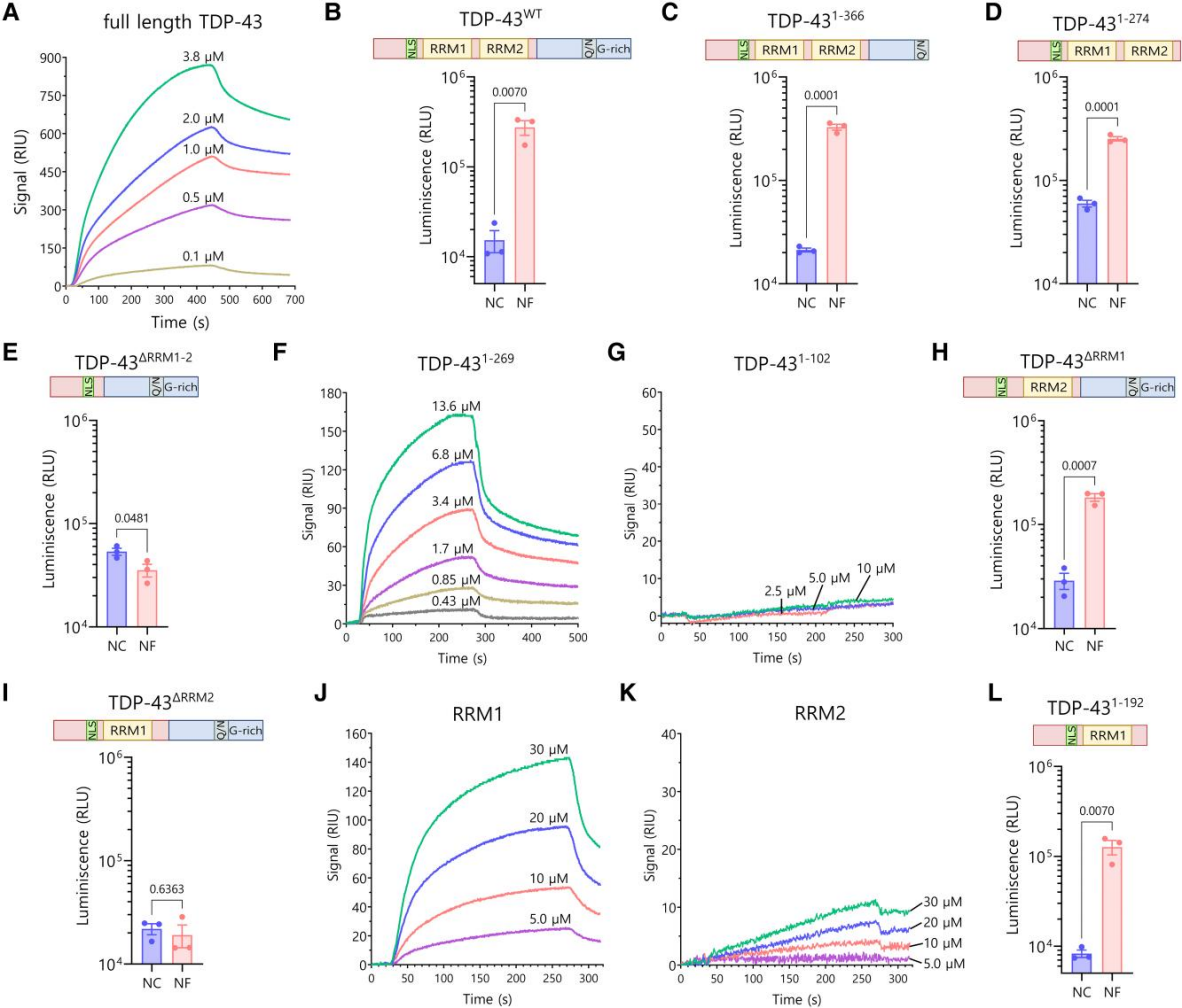
**Interaction between RGNEF/NF242 and TDP-43**. (**A**) Representative SPR sensorgrams showing the interaction between His-GST-TDP-43^wt^ (analyte) and His-MBP-RGNEF^1–275^ (ligand) at different concentrations of His-GST-TDP-43^wt^. K_D_ = 1.78 ± 0.49 μM (*n* = 4). (**B**) NanoBiT experiment showing interaction between TDP-43^wt^ (structure detailed) and NF242 (NF) (*n* = 3; NC = negative control). (**C**) NanoBiT experiment showing interaction between TDP-43^1–366^ (structure detailed) and NF242 (NF) (*n* = 3; NC = negative control). (**D**) NanoBiT experiment showing interaction between TDP-43^1–274^ (structure detailed) and NF242 (NF) (*n* = 3; NC = negative control). (**E**) NanoBiT experiment showing an absence of interaction between TDP-43^ΔRRM1–2^ (structure detailed) and NF242 (NF) (*n* = 3; NC = negative control). (**F**) Representative SPR sensorgrams showing the interaction between His-TDP-43^1–269^ (analyte) and His-MBP-RGNEF^1–275^ (ligand) at different concentrations of His-TDP-43^1–269^. K_D_ = 4.11 ± 1.33 μM (*n* = 3). (**G**) Representative SPR sensorgrams demonstrating the absence of interaction between His-TDP-43^1–102^ (analyte) and His-MBP-RGNEF^1–275^ (ligand) at different concentrations of His-TDP-43^1–102^ (*n* = 3). (**H**) NanoBiT experiment showing interaction between TDP-43^ΔRRM1^ (structure detailed) and NF242 (NF) (*n* = 3; NC = negative control). (**I**) NanoBiT experiment showing the lack of interaction between TDP-43^ΔRRM2^ (structure detailed) and NF242 (NF) (*n* = 3; NC = negative control). (**J**) Representative SPR sensorgrams demonstrating the interaction between His-RRM1 (analyte) and His-MBP-RGNEF^1–275^ (ligand) at different concentrations of His-RRM1(*n* = 4). (**K**) Representative SPR sensorgrams demonstrating weak interaction (low signal intensity) between His-RRM2 (analyte) and His-MBP-RGNEF^1–275^ (ligand) at different concentrations of His-RRM2 (*n* = 4). (**L**) NanoBiT experiment showing interaction between TDP-43^1–192^ (structure detailed) and NF242 (NF) (*n* = 3; NC = negative control). RRM = RNA recognition motif; SPR = surface plasmon resonance spectroscopy.

To further validate the interaction between NF242 and TDP-43, we measured complex formation between the two proteins directly via SPR. In this assay, NF242 was fixed to the SPR substrate as the immobilized ligand and TDP-43 was injected as the mobile analyte. To obtain sufficient amounts of purified protein for the SPR assay, we used optimized constructs of NF242 and TDP-43, which maximize recombinant expression and purification efficiency in *E. coli*. For TDP-43, we expressed the full-length wild-type protein with an N-terminal GST fusion (His-GST-TDP-43^wt^). For NF242, we included an N-terminal MBP fusion and extended the C-terminal truncation boundary of N242 by 33 residues (His-MBP- RGNEF^1–275^ or His-NF242). The SPR experiments showed direct interaction between the proteins (Fig. 1A) with a K_D_ of 1.78 ± 0.49 μM (*n* = 4).

Then, we evaluated which region of TDP-43 was critical for the interaction with NF242. For NanoBiT assays, we used a series of TDP-43 constructs with deletions of different domains of the protein with the luciferase subunits fused to the C-terminal end of both TDP-43 and NF242, having shown that these constructs showed the most significant difference to negative control between TDP-43^wt^ and NF242 (Supplementary Fig. 5H). We observed that the constructs lacking the C-terminal region of TDP-43 (TDP-43^1–366^ and TDP-43^1–274^) maintained the interaction with NF242 (Fig. 1B–D). However, when the RRM domains were removed (TDP-43^ΔRRM1–2^), no interaction was observed (Fig. 1E). SPR experiments using His-TDP-43^1–269^ as analyte confirmed the importance of the N-terminal region of TDP-43 (including both RRMs) for the direct interaction with NF242 (Fig. 1F) with a K_D_ of 4.11 ± 1.33 μM (*n* = 3). When the protein His-TDP-43^1–102^—which lacks both RRMs—was used as analyte, no interaction was detected between the proteins (Fig. 1G).

To evaluate which RRM of TDP-43 was critical for the interaction with NF242, we created two NanoBiT constructs of TDP-43 lacking RRM1 (TDP-43^ΔRRM1^) or RRM2 (TDP-43^ΔRRM2^). The deletion of RRM1 did not alter the interaction between TDP-43 and NF242 (Fig. 1H), but the deletion of RRM2 completely abolished it (Fig. 1I). SPR using His-RRM1 and His-RRM2 as ligands showed robust interaction between His-RRM1 and His-NF242 (Fig. 1J) but weak interaction with His-RRM2 (Fig. 1K). To reconcile the NanoBiT and SPR results about the role of RRM1/2 in TDP-43-NF242 interaction, we evaluated if the C-terminal region of TDP-43^ΔRRM2^ was blocking the access of NF242 to the RRM1 domain of the TDP-43 construct (Supplementary Fig. 5I). To do this, we generated a TDP-43^1–192^ NanoBiT construct, which lacks RRM2 and the C-terminal domain of the protein. Our analysis showed an interaction between TDP-43^1–192^ and NF242, confirming the blocking effect by the C-terminal domain of TDP-43 (Fig. 1L). These results suggest that both RRMs are necessary for the interaction between TDP-43 and NF242 and that RRM1 is the domain that has the strongest interaction with NF242.

**Figure 2:**
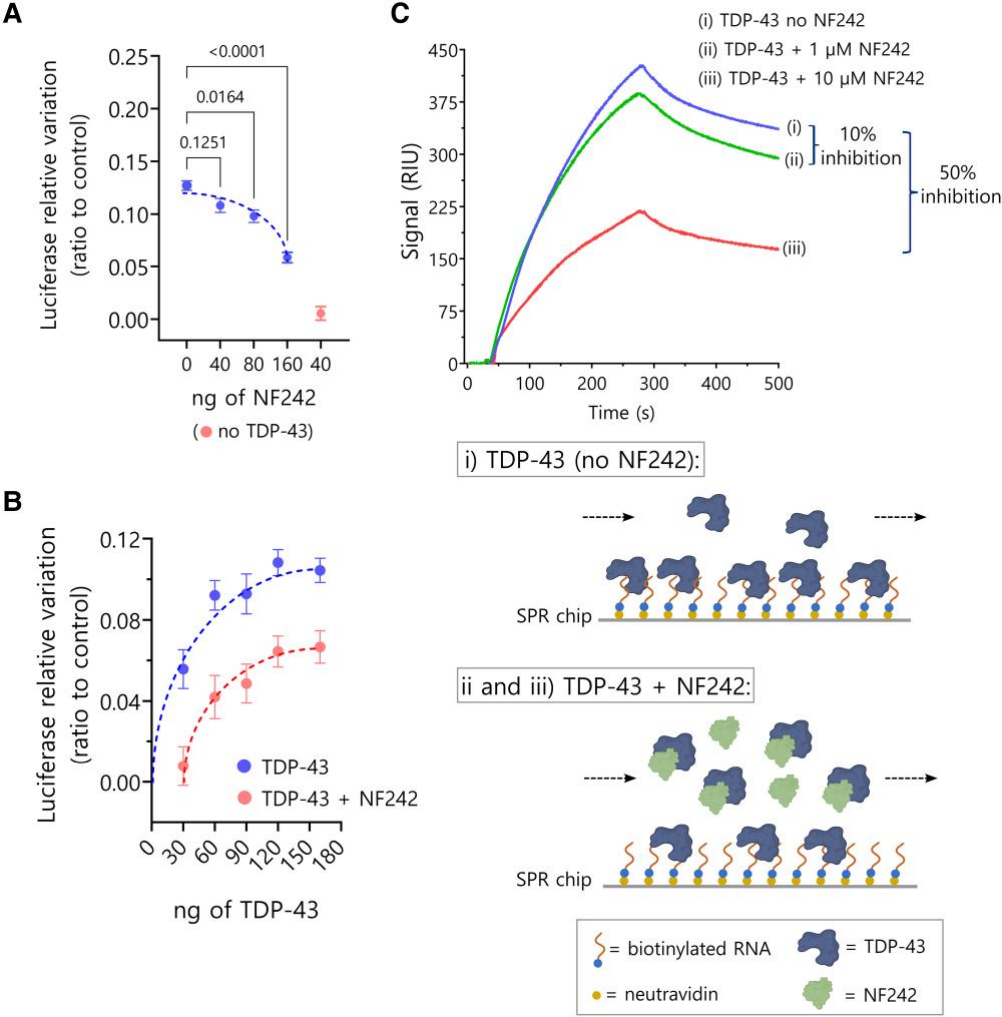
**Inhibition of TDP-43 and RNA binding by NF242**. (**A**) Luciferase assay measuring TDP-43 stabilizing activity over NEFL 3′ UTR (fixed amount of TDP-43) in presence of increasing amounts of NF242 (blue dots, *n* = 3; dotted line with associated dots). NF242 decreases TDP-43 stabilizing activity in a dose-dependent manner. The red dot (*lower right* corner outside of dotted line) shows the control in the absence of TDP-43 (*n* = 3). (**B**) Luciferase assay measuring TDP-43 stabilizing activity over NEFL 3′ UTR in presence (red; *lower* curve) of absence (blue; *upper* curve) of 120 ng of NF242 at increasing amounts of TDP-43. Displacement of the dose-response curve suggests competition of NF242 and RNA for TDP-43 (*n* = 3). (**C**) Competition experiment between NF242 and RNA for TDP-43 binding using surface plasmon resonance spectroscopy (SPR). A biotinylated RNA oligo was attached to an SPR neutravidin chip (ligand) and then 100 nM of His-TDP-43^1–269^ was used as analyte. In the accompanying schematic of the binding of His-TDP-43^1–269^ (TDP-43) to the chip (curve **i**; *upper* curve in **C**), the capability of this protein to bind RNA as observed in the sensorgram is illustrated. When His-TDP-43^1–269^ (TDP-43) and His-NF242 (NF242) were pre-incubated together at two NF242 concentrations (1 μM or 10 μM; curves **ii** and **iii**, *middle* and *lower* curves, respectively) to ensure an effect of NF242 over TDP-43, and this was injected into the SPR machine, a 10% reduction of the signal in the sensorgram (inhibition) was observed at 1 μM NF242 and 50% at 10 μM NF242 indicating that an important fraction of TDP-43 was bound to NF242 and not interacting with RNA. This confirms that NF242 blocks the access of TDP-43 to the RNA on the chip through its binding to the same site that binds RNA in TDP-43. Panel **C**(**i**–**iii**) created with BioRender.com.

To study whether the interaction with NF242 had any functional consequence for TDP-43, we used a luciferase assay that we previously developed to evaluate the regulation of the stability of *NEFL* 3′ UTR by TDP-43.^12^ We observed a moderate inhibition of NF242 over the RNA stabilizing activity of TDP-43, suggesting a competitive effect between NF242 and RNA for TDP-43 (Fig. 2A and B).

**Figure 3:**
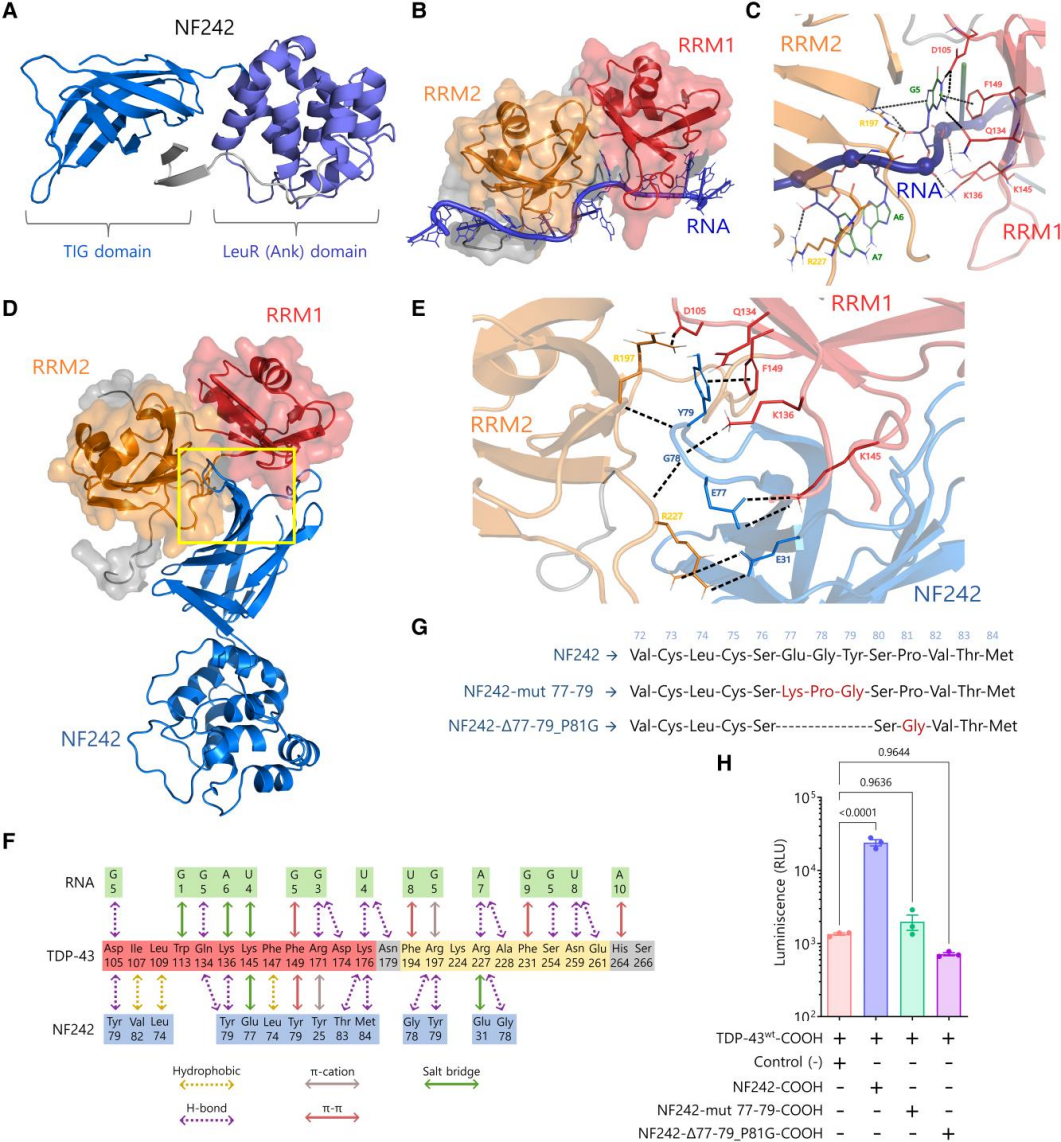
**Modelling of TDP-43-NF242 interaction**. (**A**) NF242 structure based in the atomic coordinates of RGNEF residues 1–242 (NF242) extracted from the AlphaFold Protein structure database (accession Q8N1W1). (**B**) Minimized structure of TDP-43 in complex with AUG12 RNA from experimental NMR coordinates (PDB accession: 4BS2). (**C**) Region of high inter-molecular contacts occurring between TDP-43 and RNA. (**D**) Minimized structure of TDP-43 in the complex with NF242. (**E**) Region of high intermolecular contacts occurring between TDP-43 and NF242 (yellow square in **D**) showing the most important amino acid interactions from the loop 76–81 of NF242 and the interface between RRM1 and RRM2 of TDP-43. (**F**) Summary of all intermolecular contacts. (**G**) Schematic showing the mutants used to study the importance of the loop 76–81 of the TIG domain of NF242 in the interaction with TDP-43. (**H**) NanoBiT experiment showing that the mutants NF242-mut 77–79 and NF242-Δ77–79_P81G, both fused to smBiT in the C-terminal end, do not interact with TDP-43 (*P* = 0.9636 and *P* = 0.9644, respectively). The interaction with NF242 is shown as positive control (*P* < 0.0001). RRM = RNA recognition motif.

The molecular docking modelling demonstrated the importance of both RRMs of TDP-43 in the interaction with NF242 and predicted that NF242 binds to the same TDP-43 site that binds RNA (Fig. 3A–D). As we also observed a competitive effect between NF242 and RNA for TDP-43 using the luciferase assay (Fig. 2A and B), we decided to study if NF242 directly binds to the same site of TDP-43 as RNA by performing an SPR competition experiment. First, we observed that His-TDP-43^1–269^ binds an SPR chip containing bound RNA efficiently at 100 nM of concentration (Fig. 2C). Then, we observed that when His-NF242 is present, the binding of His-TDP-43^1–269^ to RNA decreases by ∼10% and 50% at 1 and 10 μM His-NF242, respectively. The decrease of the His-TDP-43^1–269^ signal in the sensorgrams indicates that His-NF242 and RNA compete for the same His-TDP-43^1–269^ binding site. If NF242 interacted with TDP-43 at a different site than the RNA, we would have observed an additive effect in the sensorgrams. If NF242 and TDP-43 did not interact, we would have observed no change in the His-TDP-43^1–269^ SPR signal. The partial inhibitory effect in the binding of His-TDP-43^1–269^ to RNA, despite the high molar ratio between His-NF242 and His-TDP-43^1–269^, is explained because of the difference in the K_D_ between the binding of His-NF242 and His-TDP-43^1–269^, which is in the μM range, and the K_D_ for the binding between RNA and TDP-43, which is the nM range.^25^

To test if the capability of TDP-43 for RNA binding was a requirement for its interaction with NF242, we evaluated by SPR the interaction between a fragment of TDP-43 containing only the RRMs (TDP-43^101–261^) without (control) or with the mutations Phe147/149/229/231Leu (TDP-43^101–261^-F4L), which has previously been shown to completely abolish the binding of RNA.^47^ We observed that TDP-43^101–261^-F4L binds NF242 similarly to the TDP-43^101–261^ control (Supplementary Fig. 6A and B), suggesting that the capability of binding RNA by TDP-43 is not necessary for its interaction with NF242.

The *in silico* analysis suggested that the amino acids 76 to 81, corresponding to a loop in the IPT/TIG (immunoglobulin, plexins, transcription factors-like/transcription factor immunoglobulin) domain (amino acids 1 to 95) of NF242 (Supplementary Fig. 4A) are critical for the interaction with TDP-43 in the interface between the RRM1 and RRM2 domains (Fig. 3E and F). To test this, we generated two NanoBiT constructs with mutations in the loop region (Fig. 3G). We observed no interaction between NF242 and TDP-43 when the loop 76–81 of NF242 was disrupted (Fig. 3H).

Taken together, these results, obtained using two different and complementary techniques, one evaluating protein-protein interaction in living cells (NanoBiT) and the other analysing the kinetic of interaction with purified proteins *in vitro* (SPR), suggest that the interaction between NF242 and TDP-43 could have a physiological effect *in vivo* using ALS animal models.

### NF242 and TDP-43 co-expression in flies

After previously determining that RGNEF has a protective effect in cells under stress,^21^ we sought to evaluate whether RGNEF exerts this protection when TDP-43 is overexpressed. We co-transfected HEK293T cells with plasmids expressing RGNEF and TDP-43^wt^ and observed that RGNEF decreased TDP-43-induced cytotoxicity compared to TDP-43^wt^ overexpression alone (Supplementary Fig. 7A). The same result was obtained when NF242 and TDP-43^wt^ were co-expressed (Supplementary Fig. 7B). This observation and the direct interaction between NF242 and TDP-43 led us to study the *in vivo* effect of the co-expression of either NF242 or RGNEF with TDP-43^wt^. We created lines of transgenic *Drosophila melanogaster* (fruit flies) co-expressing RGNEF and TDP-43^wt^ or NF242 and TDP-43^wt^ using the UAS-GAL4 system.^48^ As neuropathological TDP-43 positive control for the experiment, we created the *GFP;TDP-43^wt^* fly, which incorporated GFP under the UAS promoter to compare only double transgenic flies and account for any possible effect caused by GAL4 acting over two UAS promoters (Supplementary Fig. 8).

**Figure 4:**
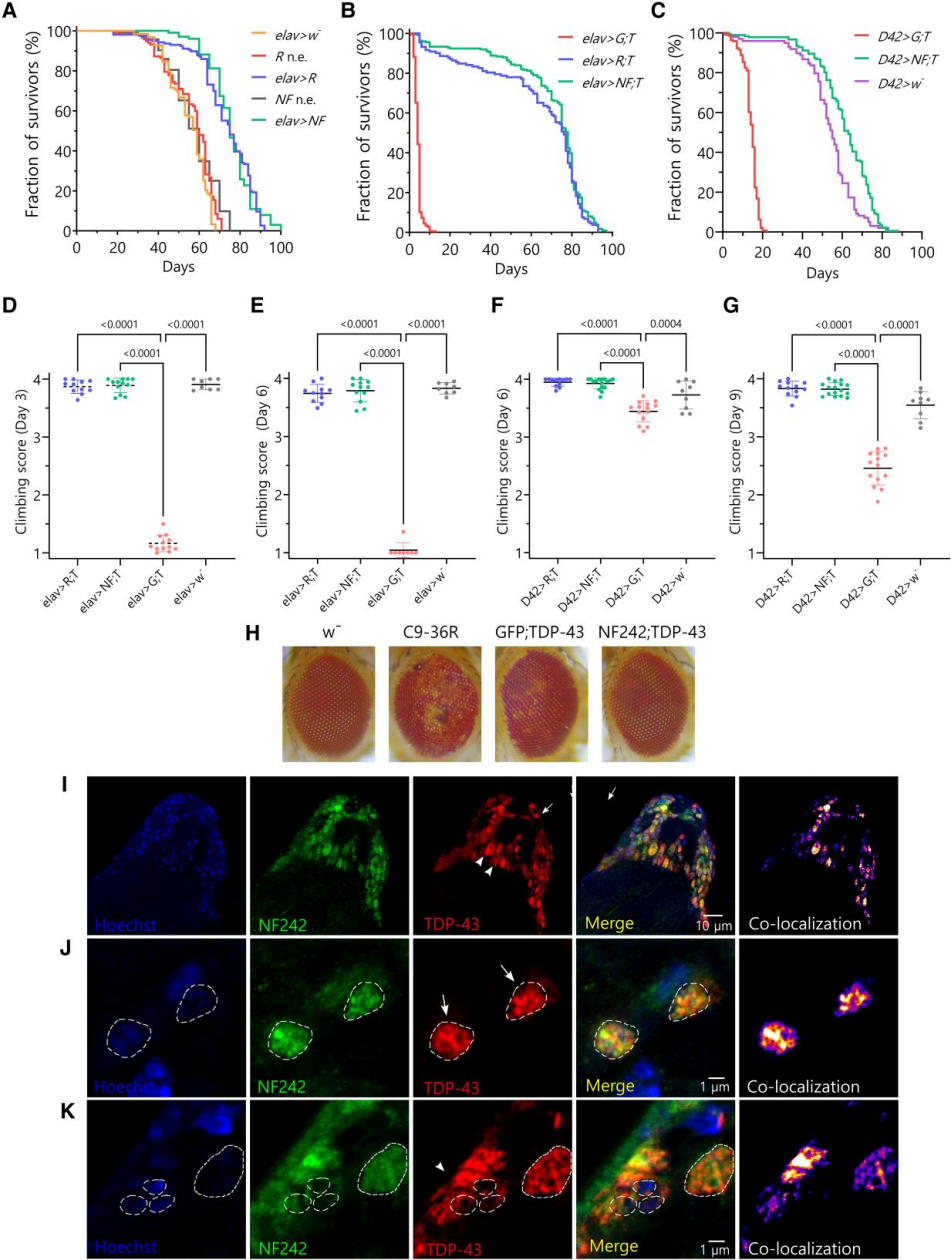
**Co-expression of RGNEF or NF242 with TDP-43 in fruit flies**. (**A**) Kaplan-Meier graph showing the survival of *elav>RGNEF* (*elav>R*; *n* = 156), *elav>NF242* (*elav>NF*; *n* = 101) *RGNEF* no driver control (*R* n.e.; *n* = 117), NF242 no driver control (*NF* n.e.; *n* = 93) and *elav>w−* (control of driver crossed with parental line; *n* = 123). The *elav>RGNEF* line shows an increased lifespan compared to *RGNEF* no driver control (*P* = 0.0035) and *elav*>*w*− (*P* < 0.0001) lines. The *elav>NF242* line shows an increased lifespan compared to *NF242* no driver control (*P* < 0.0001) and *elav>w−* (*P* < 0.0001) lines. (**B**) Kaplan-Meier graph showing the survival of *elav>GFP;TDP-43^wt^* (*elav>G; T*; *n* = 178), *elav>RGNEF;TDP-43^wt^* (*elav>R; T*, *n* = 132) and *elav>NF242;TDP-43^wt^* (*elav>NF; T*, *n* = 224). The *elav>GFP;TDP-43^wt^* line shows a reduced lifespan, an effect that is suppressed in the *elav>RGNEF; TDP-43^wt^* (*P* < 0.0001) and *elav>NF242;TDP-43^wt^* (*P* < 0.0001) lines. (**C**) Kaplan-Meier graph showing the survival of *D42>GFP; TDP-43^wt^* (*D42>G; T*; *n* = 143), *D42>NF242;TDP-43^wt^* (*D42>NF; T*, *n* = 181) and *D42>w^−^* (control of driver crossed with parental line, *n* = 98). The *D42>GFP;TDP-43^wt^* line shows a reduced lifespan, an effect that is suppressed in the *D42>NF242* line (*P* < 0.0001). The latter also show an increase in lifespan compared to the control *D42>w^−^* line (*P* < 0.0001). (**D** and **E**) Negative geotaxis assay showing the climbing score at Days 3 and 6 for *elav>RGNEF;TDP-43^wt^* (*elav>R; T*, *n* = 11; 110 flies), *elav>NF242;TDP-43^wt^* (*elav>NF; T*, *n* = 12, 120 flies), *elav>GFP;TDP-43^wt^* (*elav>G; T*; with *n* = 12; 120 flies at Day 1) and *elav>w^−^* (*n* = 8; 80 flies) lines. The *elav>GFP;TDP-43^wt^* line shows a severe motor phenotype that is suppressed when RGNEF or NF242 is co-expressed with TDP-43^wt^ in neurons (*P* < 0.0001). (**F** and **G**) Negative geotaxis assay showing the climbing score at Days 6 and 9 for *D42>RGNEF;TDP-43^wt^* (*D42>R; T*, *n* = 12; 120 flies) and *D42>NF242;TDP-43^wt^ (D42>NF; T*, *n* = 16, 160 flies), *D42>GFP;TDP-43^wt^* (*D42>G; T*; *n* = 14; 140 flies) and *D42>w^−^* (*n* = 9; 90 flies). The *D42>GFP; TDP-43^wt^* line shows a significant motor phenotype that is suppressed when RGNEF or NF242 is co-expressed with TDP-43^wt^ in motor neurons (*P* < 0.0001). (**H**) Representative images showing the eye phenotype of *GMR>w^−^* (negative control), *GMR>36R* (positive control), *GMR>GFP;TDP-43^wt^* and *GMR>NF242;TDP-43^wt^* lines. NF242 co-expression with TDP-43^wt^ suppresses the eye degeneration observed in the *GMR>NF242;TDP-43^wt^* line. (**I**) Immunofluorescence of adult *elav>NF242;TDP-43^wt^* fly brain tissue showing the co-localization between NF242 and TDP-43^wt^ in neurons. (**J** and **K**) Confocal images at higher magnification of adult *elav>NF242;TDP-43^wt^* fly brain tissue showing the co-aggregation between NF242 and TDP-43^wt^ in neurons. Nuclei are indicated with dashed lines. Arrows show nuclear co-localization and arrowheads cytoplasmic co-localization.

First, we analysed the effect of the expression of RGNEF and NF242 alone on the lifespan of the flies. When RGNEF was overexpressed using the elav pan-neuronal driver (*elav*>*RGNEF* line), we observed an increased lifespan of the flies (average of 72.89 ± 1.22 days) compared with the heterozygous driver alone control *elav*>*w^−^* (*elav* crossed with the *w*^−^ line) (average of 54.95 ± 0.88 days; *P* < 0.0001; *w*^−^ is the parental line for the transgenic flies) and the *RGNEF* line without the driver (average of 56.24 ± 1.01 days; *P* = 0.0035; Fig. 4A). When NF242 was overexpressed using the same driver (*elav*>*NF242* line), we also observed an increased lifespan of the flies (average of 75.66 ± 1.08 days) compared with the control *elav>w^−^* (*P* < 0.0001; w^−^) and the *NF242* line without the driver (average of 57.26 ± 1.24 days; *P* < 0.0001; Fig. 4A). Then, we evaluated the effect of the co-expression of RGNEF or NF242 with TDP-43 (*elav>RGNEF;TDP-43^wt^* and *elav>NF242;TDP-43^wt^* lines) on the flies’ lifespan. When compared with the *elav>GFP;TDP-43^wt^* line, which had a short lifespan (average of 4.27 ± 0.13 days) consistent with previous reports,^49-51^ both *elav>RGNEF;TDP-43^wt^* and *elav>NF242;TDP-43^wt^* lines showed a significantly longer lifespan (average of 63.73 ± 2.25 and 69.56 ± 1.44 days, respectively; *P* < 0.0001; Fig. 4B). We obtained similar results using the D42 motor neuron driver; *D42>GFP;TDP-43^wt^* line had a significantly shorter lifespan (average 14.13 ± 0.31 days) than *D42>NF242;TDP-43^wt^* line and the heterozygous driver alone control *D42>w^−^* line (average 61.07 ± 1.06 and 53.32 ± 1.44 days, respectively; *P* < 0.0001; Fig. 4C).

The effect of RGNEF and NF242 expression in the motor phenotype induced by TDP-43^wt^ in flies was evaluated using a negative geotaxis assay.^52,53^ We observed that the toxic motor phenotype induced by TDP-43^wt^ under the neuron-specific elav driver (*elav>GFP;TDP-43^wt^* line) was suppressed by either RGNEF (*elav>RGNEF;TDP-43^wt^* line) or NF242 (*elav>NF242;TDP-43^wt^* line; Fig. 4D and E). Analogous results were observed when these proteins were expressed only in motor neurons using the D42 driver (Fig. 4F and G). The effect of TDP-43^wt^, RGNEF and NF242 expression in the induction of eye degeneration was studied using the eye-specific GMR driver. *GMR>GFP;TDP-43^wt^* line showed eye degeneration as expected,^53^ but at a lesser extent than our positive control line expressing 36 C9orf72 expanded repeats^54,55^ (*GMR>C9-36R*). Neither the negative control *GMR>w^−^* nor the double transgenic line *GMR>NF242;TDP-43^wt^* demonstrated evidence of eye degeneration (Fig. 4H). *GMR>RGNEF;TDP-43^wt^* showed an eye phenotype different from *GMR>GFP;TDP-43^wt^* or *GMR>C9-36R* flies, that was also observed in *GMR>RGNEF* flies (Supplementary Fig. 9A), which suggests that this is an effect caused by RGNEF overexpression and is not related to TDP-43^wt^ toxicity.

Next, we studied the localization of TDP-43^wt^ and NF242 in the central brain and optical lobes (Supplementary Fig. 9B) of fixed *elav>NF242;TDP-43^wt^* and *elav>GFP;TDP-43^wt^* flies, by immunofluorescence. We observed that NF242 and TDP-43^wt^ co-aggregate in neurons, both in the nucleus and the cytosol (Fig. 4I–K andSupplementary Fig. 9C and D). As expected, *elav>GFP;TDP-43^wt^* control flies showed TDP-43 pathology in neurons (Supplementary Fig. 9E and F). We did not observe differences in the TDP-43 pathology between *elav>GFP;TDP-43^wt^* and *elav>NF242;TDP-43^wt^* lines (Supplementary Fig. 9G).

These results confirm that RGNEF acts as a survival factor *in vivo* and show that RGNEF and NF242 suppress the toxic motor phenotype induced by TDP-43^wt^ in flies. Also, it suggests that the co-aggregation between NF242 and TDP-43 is critical for abolishing the toxicity generated by TDP-43 overexpression in neurons.

### Ectopic NF242 expression in TDP-43 mice

The results using flies suggested a therapeutic potential for NF242. Given this, we studied the effect of the ectopic expression of NF242 in neurons using intracerebroventricular (ICV) injections of an AAV (serotype 9) in a severe murine model of ALS (rNLS8) that expresses human TDP-43^ΔNLS^ under the regulation of a Tet-Off system.^23^ AAV9 expressing GFP was used as a control.

**Figure 5:**
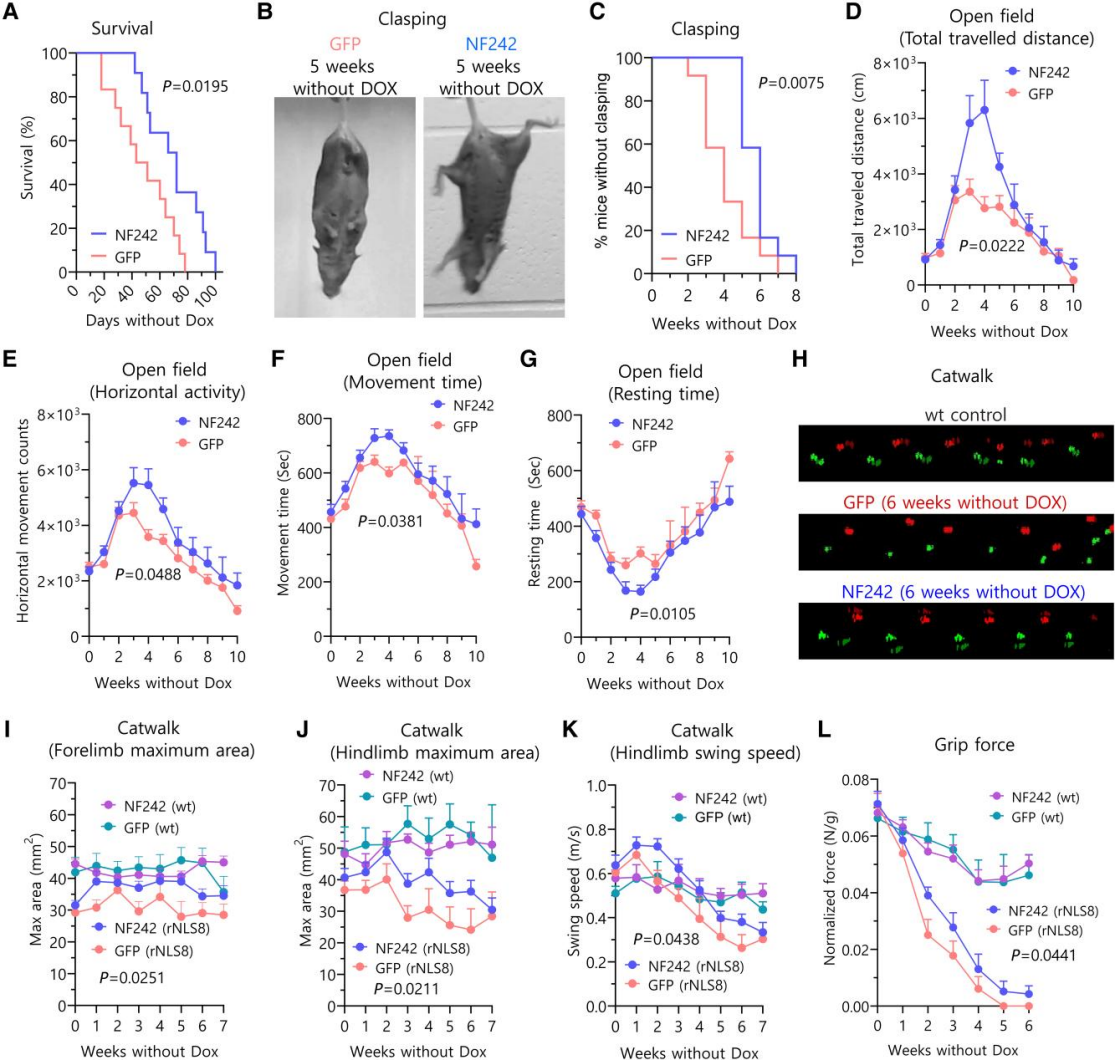
**Ectopic expression of NF242 in rNLS8 mice**. (**A**) Kaplan-Meier graph showing the increased lifespan after doxycycline (Dox) retrieval of rNLS8 mice injected with AAV9/GFP (*n* = 12) compared to mice injected with AAV9/NF242 (*n* = 11) (*P* = 0.0195). (**B**) Representative pictures showing a rNLS8 mouse injected with AAV9/GFP with clasping and a rNLS8 mouse injected with AAV9/NF242 after 5 weeks without Dox. (**C**) Kaplan-Meier graph showing clasping quantification of rNLS8 mice injected with AAV9/GFP (*n* = 12) or AAV9/NF242 (*n* = 12). AAV9/NF242 injected rNLS8 mice show a significant delay in clasping occurrence (*P* = 0.0075). (**D**–**G**) Open field test comparing rNLS8 mice injected with AAV9/GFP (*n* = 12) or AAV9/NF242 (*n* = 12). AAV9/NF242 injected rNLS8 mice show an increase in (**D**) total distance travelled (*P* = 0.0222), (**E**) horizontal activity (*P* = 0.0488), (**F**) movement time (*P* = 0.0105) and (**G**) a decrease in resting time (*P* = 0.0105). (**H**) Representative visualizations of gait assessment (Catwalk) that compares the improved gait pattern of an AAV9/NF242 injected rNLS8 mouse with an AAV9/GFP injected rNLS8 mouse at 6 weeks without Dox. Wild-type mouse shows normal gait. (**I**–**K**) Catwalk quantification comparing rNLS8 and wild-type (wt) mice injected with AAV9/GFP or AAV9/NF242 (*n* = 12 for each group of rNLS8 mice; *n* = 6 for each group of wild-type mice). The AAV9/NF242 injected rNLS8 mice show an improvement in (**I**) the forelimb maximum area (*P* = 0.0251), (**J**) the hindlimb maximum area (*P* = 0.0211) and (**K**) the hindlimb swing speed (*P* = 0.0438). (**L**) Grip force experiment showing that the AAV9/NF242 injected rNLS8 mice (*n* = 12) have a slight increase in the force compared to the AAV9/GFP injected mice (*n* = 12) (*P* = 0.0441). Wild-type mice injected with AAV9/GFP (*n* = 6) or AAV9/NF242 (*n* = 6) are shown as healthy grip force controls. GFP = green fluorescent protein.

The rNLS8 mice expressing NF242 showed a significantly longer lifespan compared with the GFP-expressing animals (NF242 average: 70.28 ± 6.12 days; GFP average: 47.92 ± 6.17 days; *P* = 0.0195; Fig. 5A). We also observed that mice injected with AAV9/NF242 had a significant improvement in clasping occurrence (NF242 average: 5.83 ± 0.27 weeks; GFP average: 4.08 ± 0.41 weeks; *P* = 0.0075; Fig. 5B and C). As the animals injected with AAV9/NF242 were visibly more active and healthier (less kyphosis and tremor, more hydrated) compared to the mice injected with AAV9/GFP (Supplementary Videos 1–4) in the first 5–6 weeks after Dox was removed from the drinking water, we next quantified locomotor activity using an open field test.^56^ Mice injected with AAV9/NF242 showed an improvement in most of the parameters evaluated for this test, including total distance travelled (Fig. 5D), horizontal activity (Fig. 5E), movement time (Fig. 5F) and resting time (Fig. 5G). Vertical activity was not different between the groups (Supplementary Fig. 10A). To assess motor function and coordination, we used gait analysis (catwalk).^57,58^ We observed that mice injected with AAV9/NF242 had an improved gait phenotype (Fig. 5H) and better results in the maximum area for fore and hindlimbs with values closer to the wild-type controls and significantly different from the AAV9/GFP injected rNLS8 mice (Fig. 5I and J). Additionally, swing speed for hindlimbs was significantly different compared to the AAV9/GFP-injected mice controls (Fig. 5K). However, the assessment against wild-type controls showed a different pattern of swing speed in the transgenic mice, which is consistent with reports showing alterations in this parameter in neurodegenerative mice models with altered locomotion.^58^

We did not observe a difference for fore and hindlimbs stride length (Supplementary Fig. 10B and C) or forelimb swing speed (Supplementary Fig. 10D). When we analysed the strength of the mice using a grip force test, we also observed a better performance for mice injected with AAV9/NF242 (Fig. 5L). We did not observe a difference in the balance using the rotarod test^59^ (Supplementary Fig. 10E) or in the weight of the mice injected with AAV9/NF242 compared to AAV9/GFP (Supplementary Fig. 10F).

**Figure 6:**
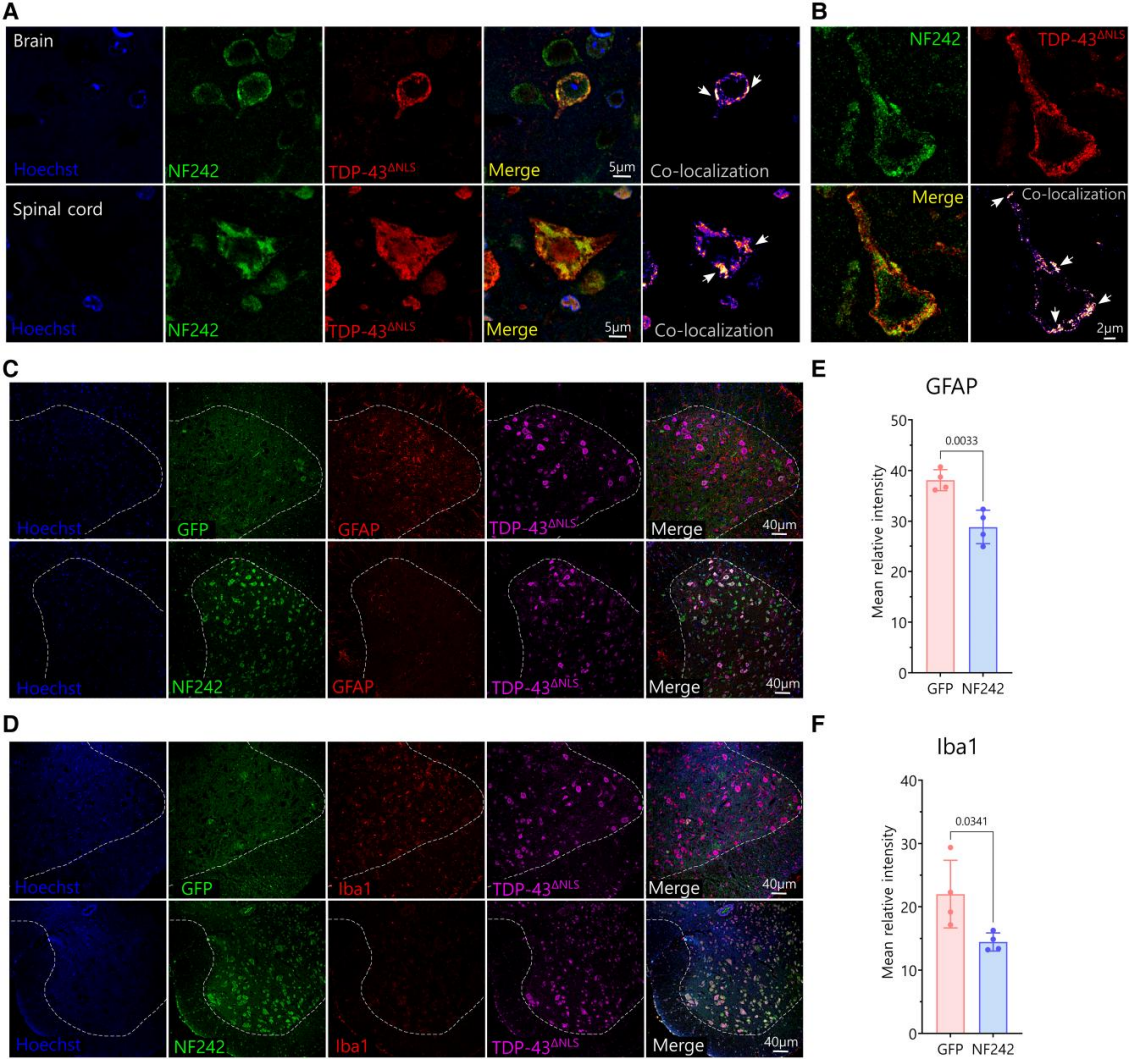
**Pathology of rNLS8 mice expressing ectopic NF242 at Week 3**. (**A**) High magnification confocal images showing the co-localization and co-aggregation (indicated by white arrows) between NF242 and TDP-43^ΔNLS^ in the brain cortex (cortical layer II–III) and spinal cord of a rNLS8 mouse injected with AAV9/NF242 after 3 weeks without doxycycline (Dox). (**B**) Super-resolution stimulated emission depletion (STED) microscopy images showing in detail the co-aggregation (indicated by white arrows) between NF242 and TDP-43^ΔNLS^ in the brain cortex (cortical layer II–III) of a rNLS8 mouse injected with AAV9/NF242 after 3 weeks without Dox. (**C** and **D**) Representative immunofluorescences of rNLS8 mice injected with AAV9/GFP and AAV9/NF242 showing the decrease in the amount of glial fibrillary acidic protein (GFAP) (**C**) and Iba1 (**D**) in the spinal cord. The anterior grey horn is separated from the white matter by a dashed white line. (**E** and **F**) Quantification showing the reduction of the levels of GFAP (**E**, *P* = 0.0033) and Iba1 (**F**, *P* = 0.0341) in the ventral horns of the lumbar spinal cord of rNLS8 mice injected with AAV9/GFP and AAV9/NF242, after 3 weeks without Dox (*n* = 4). GFP = green fluorescent protein.

Fluorescence staining showed that the AAV9/GFP and AAV9/NF242 were efficiently transduced in the brain of wild-type mice (Supplementary Fig. 11A and B). The same was observed when the viruses were injected in rNLS8 mice (Supplementary Fig. 11C and D). When we analysed the spinal cord of rNLS8 mice injected with AAV9/NF242, we observed a high efficiency of transduction (Supplementary Fig. 11E). Neurons expressing NF242 in the spinal cord and brain cortex of rNLS8 mice observed at high magnification with confocal microscopy or using STED microscopy showed extensive co-localization and co-aggregation with TDP-43^ΔNLS^ (Fig. 6A and B). When we analysed the expression of the neuroinflammatory markers in spinal cord, we observed a significant reduction in the levels of the astrogliosis marker GFAP (Fig. 6C; reduction of 59.9%; *P* = 0.0033; Fig. 6E) and the microgliosis marker Iba1 (Fig. 6D; reduction of 74.4%; *P* = 0.0341; Fig. 6F) in the NF242 expressing mice.

These results demonstrate that the ectopic expression of NF242 in neurons of mice brains and spinal cords using AAVs improves the lifespan and motor phenotype of a severe and fast-deteriorating model of ALS based on TDP-43 dysregulation.

## Discussion

Here, we show that the pathological phenotype induced by TDP-43 overexpression is suppressed in flies and ameliorated in mice by an N-terminal fragment of the RNA-binding protein RGNEF/p190RhoGEF (NF242). This is the first report of a protein fragment that is able to bind TDP-43 and has a therapeutic effect that includes improvement of motor phenotype, increased lifespan and reduction of neuroinflammatory markers in a murine ALS model.

**Figure 7:**
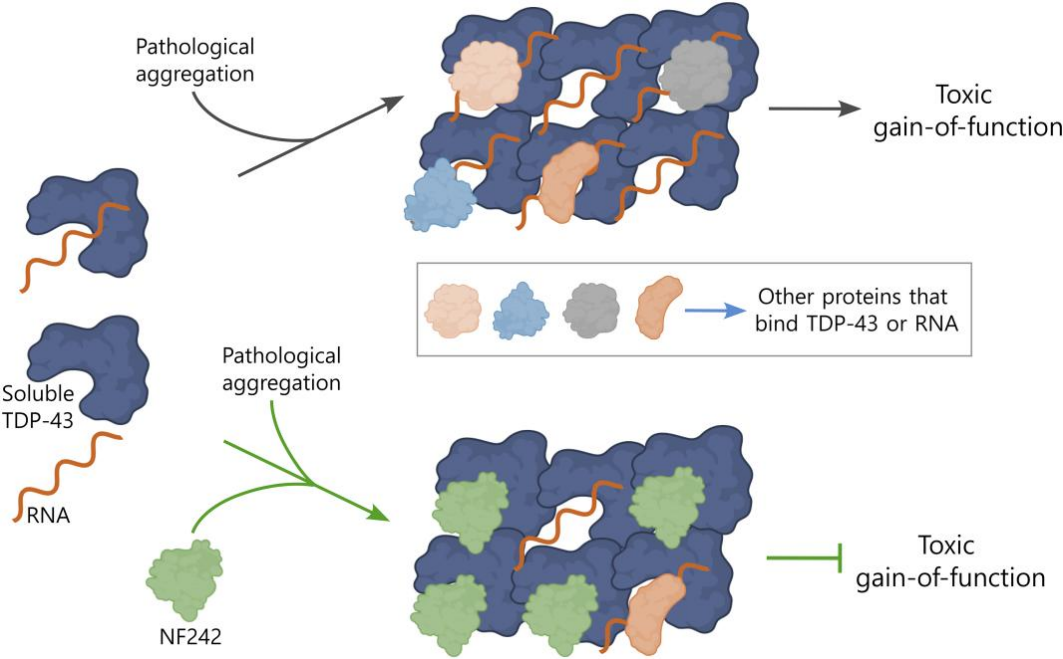
**Schematic of the proposed mechanism of action for NF242**. We hypothesize that under pathological conditions, TDP-43 aggregates sequester RNAs, other RNA-binding proteins or other proteins that can interact with TDP-43, leading to a toxic gain-of-function. When NF242 is expressed genetically or using adeno-associated viruses, it binds to TDP-43 aggregates and blocks sequestration of RNAs and proteins, thus inhibiting the toxic gain-of-function. Created with BioRender.com.

Our results support that the interaction and specific co-aggregation between NF242 and TDP-43 are key to the protective effect of NF242 against the toxicity induced by TDP-43^wt^ and TDP-43^ΔNLS^ in two *in vivo* models. As NF242 competes with RNA for the binding site of TDP-43, NF242 might be blocking the toxic gain-of-function of TDP-43 generated by sequestering RNA and other proteins into the aggregates.^11,60^ The evidence that supports this mechanism includes: (i) the confirmation that the predicted 77–79 loop of NF242 is critical for NF242-TDP-43 interaction using mutagenesis and protein-protein interaction assays (NanoBiT) in living cells (Fig. 3H); (ii) the competitive effect observed in living cells of NF242 over TDP-43 RNA stabilizing activity (Fig. 2A and B); (iii) the *in vitro* competition assay that demonstrated that NF242 directly competes with RNA for TDP-43 binding (Fig. 2C); (iv) our previous observation that under metabolic stress NF242 has a high propensity to co-aggregate with TDP-43 inclusions^22^; and (v) the robust effect of NF242 in *in vivo* models of TDP-43 pathology without deleterious consequences in controls. The latter suggests that NF242 has a higher affinity for pathological TDP-43 than for soluble TDP-43 and is supported by the observation that high amounts of NF242 are needed to inhibit RNA binding to soluble TDP-43. In our model, we propose that this preference of NF242 for pathological TDP-43 leads to co-aggregation and blockage of the TDP-43’s gain of toxic function (Fig. 7). The relevance of our findings is that it is not necessary to eliminate the aggregates to obtain a therapeutic effect. Rather, blocking toxic aggregates with an innocuous protein (co-aggregating) could be as beneficial as eliminating the aggregates.

Considering the evidence we have shown of the interaction between NF242 and TDP-43, it is intriguing that, to date, RGNEF has not been found as an interactor of TDP-43 in proteomic analysis. Technical reasons could explain this absence. There are four studies that have systematically described TDP-43 interactors. Two of them might have not detected RGNEF because they were based on immunoprecipitation/pull-down from cell lines with low RGNEF expression.^61,62^ The other two, because two-hybrid assays were performed and RGNEF was not included in the analysed library.^63,64^ In general, TDP-43 and RGNEF are observed in different subcellular compartments and they co-aggregate only under pathological conditions.^11,12,22^ More recently, it has been shown that 95 genes are co-regulated by TDP-43 and RGNEF, including axonal guidance genes (specifically *SRGAP3*, *MPPED2*, *GREM2* and *CFL2*), through regulation of the rate of long-intron processing,^65^ which suggests that these proteins have complementary functions under basal conditions.

Thus far, the RRMs of TDP-43 have been targeted for potential therapeutic approaches in a few *in vitro* and *in vivo* studies using flies and mice. This includes studies with small molecules, such as compounds containing extended planar aromatic moieties,^66^ ATP,^67^ the chemical rTRD01^68^ and an antibody against the RRM1 of TDP-43.^69^ These data support our findings demonstrating that targeting the RRM domains of TDP-43 can improve the phenotype of TDP-43 proteinopathies.

In the rNLS8 mice, a well studied ALS animal model,^23,70-72^ the mitigation effect of the AAV9/NF242 over the motor phenotype, lasted 5–6 weeks on average. The progression of signs thereafter could be explained by two reasons. First, the high and permanent expression of TDP-43^ΔNLS^ in the model and the spreading of the TDP-43 pathology beyond the cells expressing NF242. Specifically, we observed a significant increase of TDP-43 pathology in the cortical layer I at 6 weeks without Dox in rNLS8 mice injected with AAV9/NF242. This increase of TDP-43 pathology was correlated with an increase of glial fibrillary acidic protein (GFAP) in the cortical layers I and II–III and an extensive co-localization of TDP-43^ΔNLS^ with GFAP mainly in the cortical layer I (Supplementary Fig. 12), suggesting the spreading of TDP-43 pathology to astrocytes after several weeks of TDP-43^ΔNLS^ neuronal expression. Second, the suppression of the endogenous murine TDP-43 expression in the rNLS8 mice^23^; the loss-of-function is an inherent pathological factor in the model that cannot be accounted for by our therapeutic approach.

Considering that the rNLS8 mice have previously shown resistance to other therapeutic approaches, such as riluzole treatment,^24^ MMP-9 reduction^73^ or miR-23a suppression,^74^ the more encouraging evidence that we show in this work highlights the therapeutic potential of our approach after modifying the phenotype of this severe murine model of ALS.

In conclusion, our study suggests that a therapeutic strategy expressing NF242 or a biologically active fragment of NF242 could be promising in humans affected by TDP-43 proteinopathies. While in this study we described a critical region of NF242 for the interaction with TDP-43, further work is needed to reduce the size of the protein used while maintaining its biological activity without altering its structural conformation. The fact that this approach uses a fragment of a protein already expressed in humans, suggests that the secondary effects associated with the use of therapeutic antibodies^75,76^ could be minimized or avoided.

A potential treatment using this TDP-43’s gain-of-function targeting approach might need to be combined with drugs that target its loss-of-function, and potentially with drugs focused on different targets, such as autophagy.^77^ Currently, it seems we are on the edge of a new era for the developing of treatments for neurodegenerative diseases such as ALS and FTD.

## Supplementary Material

awae078_Supplementary_Data

## Data Availability

The authors confirm that the data supporting the findings of this study are available within the article and/or its Supplementary material.

## References

[1] Strong MJ , Kesavapany S, Pant HC. The pathobiology of amyotrophic lateral sclerosis: A proteinopathy? J Neuropathol Exp Neurol. 2005;64:649–664. 16106213 10.1097/01.jnen.0000173889.71434.ea

[2] Corcia P , Beltran S, Bakkouche SE, Couratier P. Therapeutic news in ALS. Rev Neurol (Paris). 2021;177:544–549. 33781562 10.1016/j.neurol.2020.12.003

[3] Droppelmann CA , Campos-Melo D, Ishtiaq M, Volkening K, Strong MJ. RNA metabolism in ALS: When normal processes become pathological. Amyotroph Lateral Scler Frontotemporal Degener. 2014;15(5–6):321–336. 24555412 10.3109/21678421.2014.881377

[4] Arai T , Hasegawa M, Akiyama H, et al TDP-43 is a component of ubiquitin-positive tau-negative inclusions in frontotemporal lobar degeneration and amyotrophic lateral sclerosis. Biochem Biophys Res Commun. 2006;351:602–611. 17084815 10.1016/j.bbrc.2006.10.093

[5] Neumann M , Sampathu DM, Kwong LK, et al Ubiquitinated TDP-43 in frontotemporal lobar degeneration and amyotrophic lateral sclerosis. Science. 2006;314:130–133. 17023659 10.1126/science.1134108

[6] Kwiatkowski TJ, Jr., Bosco DA, Leclerc AL, et al Mutations in the FUS/TLS gene on chromosome 16 cause familial amyotrophic lateral sclerosis. Science. 2009;323:1205–1208. 19251627 10.1126/science.1166066

[7] Vance C , Rogelj B, Hortobagyi T, et al Mutations in FUS, an RNA processing protein, cause familial amyotrophic lateral sclerosis type 6. Science. 2009;323:1208–1211. 19251628 10.1126/science.1165942 PMC4516382

[8] Couthouis J , Hart MP, Shorter J, et al A yeast functional screen predicts new candidate ALS disease genes. Proc Natl Acad Sci U S A. 2011;108:20881–20890. 22065782 10.1073/pnas.1109434108 PMC3248518

[9] Couthouis J , Hart MP, Erion R, et al Evaluating the role of the FUS/TLS-related gene EWSR1 in amyotrophic lateral sclerosis. Hum Mol Genet. 2012;21:2899–2911. 22454397 10.1093/hmg/dds116 PMC3373238

[10] Collins M , Riascos D, Kovalik T, et al The RNA-binding motif 45 (RBM45) protein accumulates in inclusion bodies in amyotrophic lateral sclerosis (ALS) and frontotemporal lobar degeneration with TDP-43 inclusions (FTLD-TDP) patients. Acta Neuropathol. 2012;124:717–732. 22993125 10.1007/s00401-012-1045-x PMC3472056

[11] Keller BA , Volkening K, Droppelmann CA, Ang LC, Rademakers R, Strong MJ. Co-aggregation of RNA binding proteins in ALS spinal motor neurons: Evidence of a common pathogenic mechanism. Acta Neuropathol. 2012;124:733–747. 22941224 10.1007/s00401-012-1035-z

[12] Droppelmann CA , Keller BA, Campos-Melo D, Volkening K, Strong MJ. Rho guanine nucleotide exchange factor is an NFL mRNA destabilizing factor that forms cytoplasmic inclusions in amyotrophic lateral sclerosis. Neurobiol Aging. 2013;34:248–262. 22835604 10.1016/j.neurobiolaging.2012.06.021

[13] Kim HJ , Kim NC, Wang YD, et al Mutations in prion-like domains in hnRNPA2B1 and hnRNPA1 cause multisystem proteinopathy and ALS. Nature. 2013;495:467–473. 23455423 10.1038/nature11922 PMC3756911

[14] Gao J , Wang L, Huntley ML, Perry G, Wang X. Pathomechanisms of TDP-43 in neurodegeneration. J Neurochem. 2018;146:7–20. 10.1111/jnc.14327 PMC6110993 29486049

[15] Prasad A , Bharathi V, Sivalingam V, Girdhar A, Patel BK. Molecular mechanisms of TDP-43 misfolding and pathology in amyotrophic lateral sclerosis. Front Mol Neurosci. 2019;12:25. 30837838 10.3389/fnmol.2019.00025 PMC6382748

[16] Ling SC , Polymenidou M, Cleveland DW. Converging mechanisms in ALS and FTD: Disrupted RNA and protein homeostasis. Neuron. 2013;79:416–438. 23931993 10.1016/j.neuron.2013.07.033 PMC4411085

[17] Liao YZ , Ma J, Dou JZ. The role of TDP-43 in neurodegenerative disease. Mol Neurobiol. 2022;59:4223–4241. 35499795 10.1007/s12035-022-02847-x

[18] Palomo V , Tosat-Bitrian C, Nozal V, Nagaraj S, Martin-Requero A, Martinez A. TDP-43: A key therapeutic target beyond amyotrophic lateral sclerosis. ACS Chem Neurosci. 2019;10:1183–1196. 30785719 10.1021/acschemneuro.9b00026

[19] Buratti E . Targeting TDP-43 proteinopathy with drugs and drug-like small molecules. Br J Pharmacol. 2021;178:1298–1315. 32469420 10.1111/bph.15148

[20] Hayes LR , Kalab P. Emerging therapies and novel targets for TDP-43 proteinopathy in ALS/FTD. Neurotherapeutics. 2022;19:1061–1084. 35790708 10.1007/s13311-022-01260-5 PMC9587158

[21] Cheung K , Droppelmann CA, MacLellan A, et al Rho guanine nucleotide exchange factor (RGNEF) is a prosurvival factor under stress conditions. Mol Cell Neurosci. 2017;82:88–95. 28495450 10.1016/j.mcn.2017.05.003

[22] Droppelmann CA , Campos-Melo D, Moszczynski AJ, Amzil H, Strong MJ. TDP-43 aggregation inside micronuclei reveals a potential mechanism for protein inclusion formation in ALS. Sci Rep. 2019;9:19928. 31882736 10.1038/s41598-019-56483-y PMC6934605

[23] Walker AK , Spiller KJ, Ge G, et al Functional recovery in new mouse models of ALS/FTLD after clearance of pathological cytoplasmic TDP-43. Acta Neuropathol. 2015;130:643–660. 26197969 10.1007/s00401-015-1460-x PMC5127391

[24] Wright AL , Della Gatta PA, Le S, et al Riluzole does not ameliorate disease caused by cytoplasmic TDP-43 in a mouse model of amyotrophic lateral sclerosis. Eur J Neurosci. 2021;54:6237–6255. 34390052 10.1111/ejn.15422

[25] Lukavsky PJ , Daujotyte D, Tollervey JR, et al Molecular basis of UG-rich RNA recognition by the human splicing factor TDP-43. Nat Struct Mol Biol. 2013;20:1443–1449. 24240615 10.1038/nsmb.2698

[26] Varadi M , Anyango S, Deshpande M, et al AlphaFold Protein Structure Database: massively expanding the structural coverage of protein-sequence space with high-accuracy models. Nucleic Acids Res. 2022;50(D1):D439–D444. 34791371 10.1093/nar/gkab1061 PMC8728224

[27] Holm L . Dali server: Structural unification of protein families. Nucleic Acids Res. 2022;50(W1):W210–W215. 35610055 10.1093/nar/gkac387 PMC9252788

[28] Quignot C , Postic G, Bret H, et al Interevdock3: A combined template-based and free docking server with increased performance through explicit modeling of complex homologs and integration of covariation-based contact maps. Nucleic Acids Res. 2021;49(W1):W277–W284. 33978743 10.1093/nar/gkab358 PMC8265070

[29] Vajda S , Yueh C, Beglov D, et al New additions to the ClusPro server motivated by CAPRI. Proteins. 2017;85:435–444. 27936493 10.1002/prot.25219 PMC5313348

[30] Jumper J , Evans R, Pritzel A, et al Highly accurate protein structure prediction with AlphaFold. Nature. 2021;596:583–589. 34265844 10.1038/s41586-021-03819-2 PMC8371605

[31] Mirdita M , Schutze K, Moriwaki Y, Heo L, Ovchinnikov S, Steinegger M. ColabFold: Making protein folding accessible to all. Nat Methods. 2022;19:679–682. 35637307 10.1038/s41592-022-01488-1 PMC9184281

[32] Alford RF , Leaver-Fay A, Jeliazkov JR, et al The Rosetta all-atom energy function for macromolecular modeling and design. J Chem Theory Comput. 2017;13:3031–3048. 28430426 10.1021/acs.jctc.7b00125 PMC5717763

[33] Todd AM , Staveley BE. Novel assay and analysis for measuring climbing ability in Drosophila. Drosoph Inf Serv. 2004;87:101–107.

[34] Kucherenko MM , Marrone AK, Rishko VM, Yatsenko AS, Klepzig A, Shcherbata HR. Paraffin-embedded and frozen sections of Drosophila adult muscles. J Vis Exp. 2010;46:2438. 10.3791/2438 PMC3159657 21206479

[35] Li Q , Lau A, Morris TJ, Guo L, Fordyce CB, Stanley EF. A syntaxin 1, Galpha(o), and N-type calcium channel complex at a presynaptic nerve terminal: Analysis by quantitative immunocolocalization. J Neurosci. 2004;24:4070–4081. 15102922 10.1523/JNEUROSCI.0346-04.2004 PMC6729428

[36] Guyenet SJ , Furrer SA, Damian VM, Baughan TD, La Spada AR, Garden GA. A simple composite phenotype scoring system for evaluating mouse models of cerebellar ataxia. J Vis Exp. 2010;39:1787. 10.3791/1787 PMC3121238 20495529

[37] Brooks SP , Dunnett SB. Tests to assess motor phenotype in mice: A user's guide. Nat Rev Neurosci. 2009;10:519–529. 19513088 10.1038/nrn2652

[38] Castro B , Kuang S. Evaluation of muscle performance in mice by treadmill exhaustion test and whole-limb grip strength assay. Bio Protoc. 2017;7:e2237. 10.21769/BioProtoc.2237 PMC5510664 28713848

[39] Koopmans GC , Deumens R, Honig WM, Hamers FP, Steinbusch HW, Joosten EA. The assessment of locomotor function in spinal cord injured rats: The importance of objective analysis of coordination. J Neurotrauma. 2005;22:214–225. 15716628 10.1089/neu.2005.22.214

[40] Walter J , Kovalenko O, Younsi A, Grutza M, Unterberg A, Zweckberger K. The CatWalk XT(R) is a valid tool for objective assessment of motor function in the acute phase after controlled cortical impact in mice. Behav Brain Res. 2020;392:112680. 32479852 10.1016/j.bbr.2020.112680

[41] Martins-Silva C , De Jaeger X, Guzman MS, et al Novel strains of mice deficient for the vesicular acetylcholine transporter: Insights on transcriptional regulation and control of locomotor behavior. PLoS One. 2011;6:e17611. 21423695 10.1371/journal.pone.0017611 PMC3053374

[42] Alfieri JA , Silva PR, Igaz LM. Early cognitive/social deficits and late motor phenotype in conditional wild-type TDP-43 transgenic mice. Front Aging Neurosci. 2016;8:310. 28066234 10.3389/fnagi.2016.00310 PMC5167738

[43] Alfieri JA , Pino NS, Igaz LM. Reversible behavioral phenotypes in a conditional mouse model of TDP-43 proteinopathies. J Neurosci. 2014;34:15244–15259. 25392493 10.1523/JNEUROSCI.1918-14.2014 PMC4298649

[44] Solomon JA , Tarnopolsky MA, Hamadeh MJ. One universal common endpoint in mouse models of amyotrophic lateral sclerosis. PLoS One. 2011;6:e20582. 21687686 10.1371/journal.pone.0020582 PMC3110799

[45] Dixon AS , Schwinn MK, Hall MP, et al NanoLuc complementation reporter optimized for accurate measurement of protein interactions in cells. ACS Chem Biol. 2016;11:400–408. 26569370 10.1021/acschembio.5b00753

[46] Winton MJ , Igaz LM, Wong MM, Kwong LK, Trojanowski JQ, Lee VM. Disturbance of nuclear and cytoplasmic TAR DNA-binding protein (TDP-43) induces disease-like redistribution, sequestration, and aggregate formation. J Biol Chem. 2008;283:13302–13309. 18305110 10.1074/jbc.M800342200 PMC2442318

[47] Buratti E , Baralle FE. Characterization and functional implications of the RNA binding properties of nuclear factor TDP-43, a novel splicing regulator of CFTR exon 9. J Biol Chem. 2001;276:36337–36343. 11470789 10.1074/jbc.M104236200

[48] Brand AH , Perrimon N. Targeted gene expression as a means of altering cell fates and generating dominant phenotypes. Development. 1993;118:401–415. 8223268 10.1242/dev.118.2.401

[49] Voigt A , Herholz D, Fiesel FC, et al TDP-43-mediated neuron loss in vivo requires RNA-binding activity. PLoS One. 2010;5:e12247. 20806063 10.1371/journal.pone.0012247 PMC2923622

[50] Hanson KA , Kim SH, Wassarman DA, Tibbetts RS. Ubiquilin modifies TDP-43 toxicity in a Drosophila model of amyotrophic lateral sclerosis (ALS). J Biol Chem. 2010;285:11068–11072. 20154090 10.1074/jbc.C109.078527 PMC2856981

[51] Miguel L , Frebourg T, Campion D, Lecourtois M. Both cytoplasmic and nuclear accumulations of the protein are neurotoxic in Drosophila models of TDP-43 proteinopathies. Neurobiol Dis. 2011;41:398–406. 20951205 10.1016/j.nbd.2010.10.007

[52] Feany MB , Bender WW. A Drosophila model of Parkinson's disease. Nature. 2000;404:394–398. 10746727 10.1038/35006074

[53] Li Y , Ray P, Rao EJ, et al A Drosophila model for TDP-43 proteinopathy. Proc Natl Acad Sci U S A. 2010;107:3169–3174. 20133767 10.1073/pnas.0913602107 PMC2840283

[54] Mizielinska S , Gronke S, Niccoli T, et al C9orf72 repeat expansions cause neurodegeneration in Drosophila through arginine-rich proteins. Science. 2014;345:1192–1194. 25103406 10.1126/science.1256800 PMC4944841

[55] Tran H , Almeida S, Moore J, et al Differential toxicity of nuclear RNA foci versus dipeptide repeat proteins in a Drosophila model of C9ORF72 FTD/ALS. Neuron. 2015;87:1207–1214. 26402604 10.1016/j.neuron.2015.09.015 PMC4589299

[56] Tatem KS , Quinn JL, Phadke A, Yu Q, Gordish-Dressman H, Nagaraju K. Behavioral and locomotor measurements using an open field activity monitoring system for skeletal muscle diseases. J Vis Exp. 2014;91:51785. 10.3791/51785 PMC4672952 25286313

[57] Xu YF , Gendron TF, Zhang YJ, et al Wild-type human TDP-43 expression causes TDP-43 phosphorylation, mitochondrial aggregation, motor deficits, and early mortality in transgenic mice. J Neurosci. 2010;30:10851–10859. 20702714 10.1523/JNEUROSCI.1630-10.2010 PMC3056148

[58] Preisig DF , Kulic L, Kruger M, et al High-speed video gait analysis reveals early and characteristic locomotor phenotypes in mouse models of neurodegenerative movement disorders. Behav Brain Res. 2016;311:340–353. 27233823 10.1016/j.bbr.2016.04.044

[59] Wils H , Kleinberger G, Janssens J, et al TDP-43 transgenic mice develop spastic paralysis and neuronal inclusions characteristic of ALS and frontotemporal lobar degeneration. Proc Natl Acad Sci U S A. 2010;107:3858–3863. 20133711 10.1073/pnas.0912417107 PMC2840518

[60] Yang H , Hu HY. Sequestration of cellular interacting partners by protein aggregates: Implication in a loss-of-function pathology. FEBS J. 2016;283:3705–3717. 27016044 10.1111/febs.13722

[61] Freibaum BD , Chitta RK, High AA, Taylor JP. Global analysis of TDP-43 interacting proteins reveals strong association with RNA splicing and translation machinery. J Proteome Res. 2010;9:1104–1120. 20020773 10.1021/pr901076y PMC2897173

[62] Chou CC , Zhang Y, Umoh ME, et al TDP-43 pathology disrupts nuclear pore complexes and nucleocytoplasmic transport in ALS/FTD. Nat Neurosci. 2018;21:228–239. 29311743 10.1038/s41593-017-0047-3 PMC5800968

[63] Volkening K , Keller BA, Leystra-Lantz C, Strong MJ. RNA and protein interactors with TDP-43 in human spinal-cord lysates in amyotrophic lateral sclerosis. J Proteome Res. 2018;17:1712–1729. 29513014 10.1021/acs.jproteome.8b00126

[64] Haenig C , Atias N, Taylor AK, et al Interactome mapping provides a network of neurodegenerative disease proteins and uncovers widespread protein aggregation in affected brains. Cell Rep. 2020;32:108050. 32814053 10.1016/j.celrep.2020.108050

[65] Abbassi Y , Cappelli S, Spagnolo E, et al Axon guidance genes are regulated by TDP-43 and RGNEF through the rate of long-intron processing. *bioRxiv*. [Preprint] doi:10.1101/2023.12.05.570131 39360635

[66] Fang MY , Markmiller S, Vu AQ, et al Small-molecule modulation of TDP-43 recruitment to stress granules prevents persistent TDP-43 accumulation in ALS/FTD. Neuron. 2019;103:802–819.e11. 31272829 10.1016/j.neuron.2019.05.048 PMC6728177

[67] Dang M , Kang J, Lim L, Li Y, Wang L, Song J. ATP is a cryptic binder of TDP-43 RRM domains to enhance stability and inhibit ALS/AD-associated fibrillation. Biochem Biophys Res Commun. 2020;522:247–253. 31759630 10.1016/j.bbrc.2019.11.088

[68] Francois-Moutal L , Felemban R, Scott DD, et al Small molecule targeting TDP-43's RNA recognition motifs reduces locomotor defects in a Drosophila model of Amyotrophic Lateral Sclerosis (ALS). ACS Chem Biol. 2019;14:2006–2013. 31241884 10.1021/acschembio.9b00481 PMC6911355

[69] Pozzi S , Thammisetty SS, Codron P, et al Virus-mediated delivery of antibody targeting TAR DNA-binding protein-43 mitigates associated neuropathology. J Clin Invest. 2019;129:1581–1595. 30667370 10.1172/JCI123931 PMC6436898

[70] Spiller KJ , Cheung CJ, Restrepo CR, et al Selective motor neuron resistance and recovery in a new inducible mouse model of TDP-43 proteinopathy. J Neurosci. 2016;36:7707–7717. 27445147 10.1523/JNEUROSCI.1457-16.2016 PMC6705561

[71] Hunter M , Spiller KJ, Dominique MA, et al Microglial transcriptome analysis in the rNLS8 mouse model of TDP-43 proteinopathy reveals discrete expression profiles associated with neurodegenerative progression and recovery. Acta Neuropathol Commun. 2021;9:140. 34412701 10.1186/s40478-021-01239-x PMC8377972

[72] Hur SK , Hunter M, Dominique MA, et al Slow motor neurons resist pathological TDP-43 and mediate motor recovery in the rNLS8 model of amyotrophic lateral sclerosis. Acta Neuropathol Commun. 2022;10:75. 35568882 10.1186/s40478-022-01373-0 PMC9107273

[73] Spiller KJ , Khan T, Dominique MA, et al Reduction of matrix metalloproteinase 9 (MMP-9) protects motor neurons from TDP-43-triggered death in rNLS8 mice. Neurobiol Dis. 2019;124:133–140. 30458231 10.1016/j.nbd.2018.11.013 PMC7053168

[74] Tsitkanou S , Della Gatta PA, Abbott G, et al miR-23a suppression accelerates functional decline in the rNLS8 mouse model of TDP-43 proteinopathy. Neurobiol Dis. 2022;162:105559. 34774794 10.1016/j.nbd.2021.105559

[75] Hansel TT , Kropshofer H, Singer T, Mitchell JA, George AJ. The safety and side effects of monoclonal antibodies. Nat Rev Drug Discov. 2010;9:325–338. 20305665 10.1038/nrd3003

[76] Baldo BA . Immune- and non-immune-mediated adverse effects of monoclonal antibody therapy: A survey of 110 approved antibodies. Antibodies (Basel). 2022;11:17. 35323191 10.3390/antib11010017 PMC8944650

[77] Djajadikerta A , Keshri S, Pavel M, Prestil R, Ryan L, Rubinsztein DC. Autophagy induction as a therapeutic strategy for neurodegenerative diseases. J Mol Biol. 2020;432:2799–2821. 31887286 10.1016/j.jmb.2019.12.035

