# Supplementary material for: Mitigation of TDP-43 toxic phenotype by an RGNEF fragment in amyotrophic lateral sclerosis models
Source: Brain. 2024 May 13;147(6):2053–68. doi: 10.1093/brain/awae078 (PMC11146434; doi:10.1093/brain/awae078)
Supplement: awae078_Supplementary_Data [file awae078_supplementary_data.zip › brain-2023-01760-File009.pdf]

Supplementary material

Supplementary Figure 1

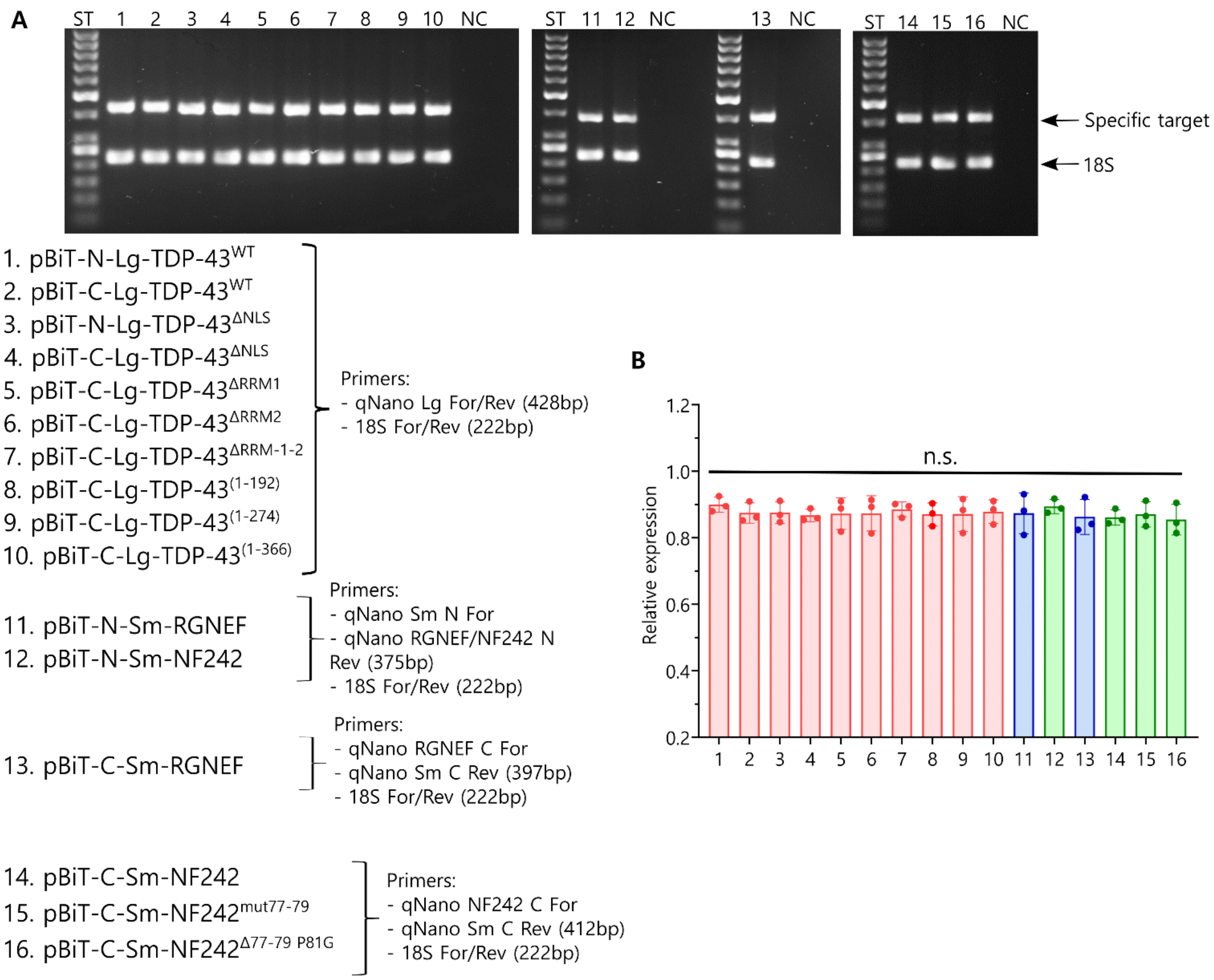

Supplementary Fig. 1 | Expression control of NanoBiT constructs. Semi-quantitative PCR showing the expression of the NanoBiT constructs used in this work. **A**, agarose gel showing the amplification of the NanoBiT specific targets and 18S used as reference gen. The constructs and the primers used are detailed in the figure. **B**, Graph showing the quantification of the expression as the ratio to 18S for each specific target. The statistical analysis didn't show a significant difference in the expression levels between all the constructs.

Supplementary Figure 2

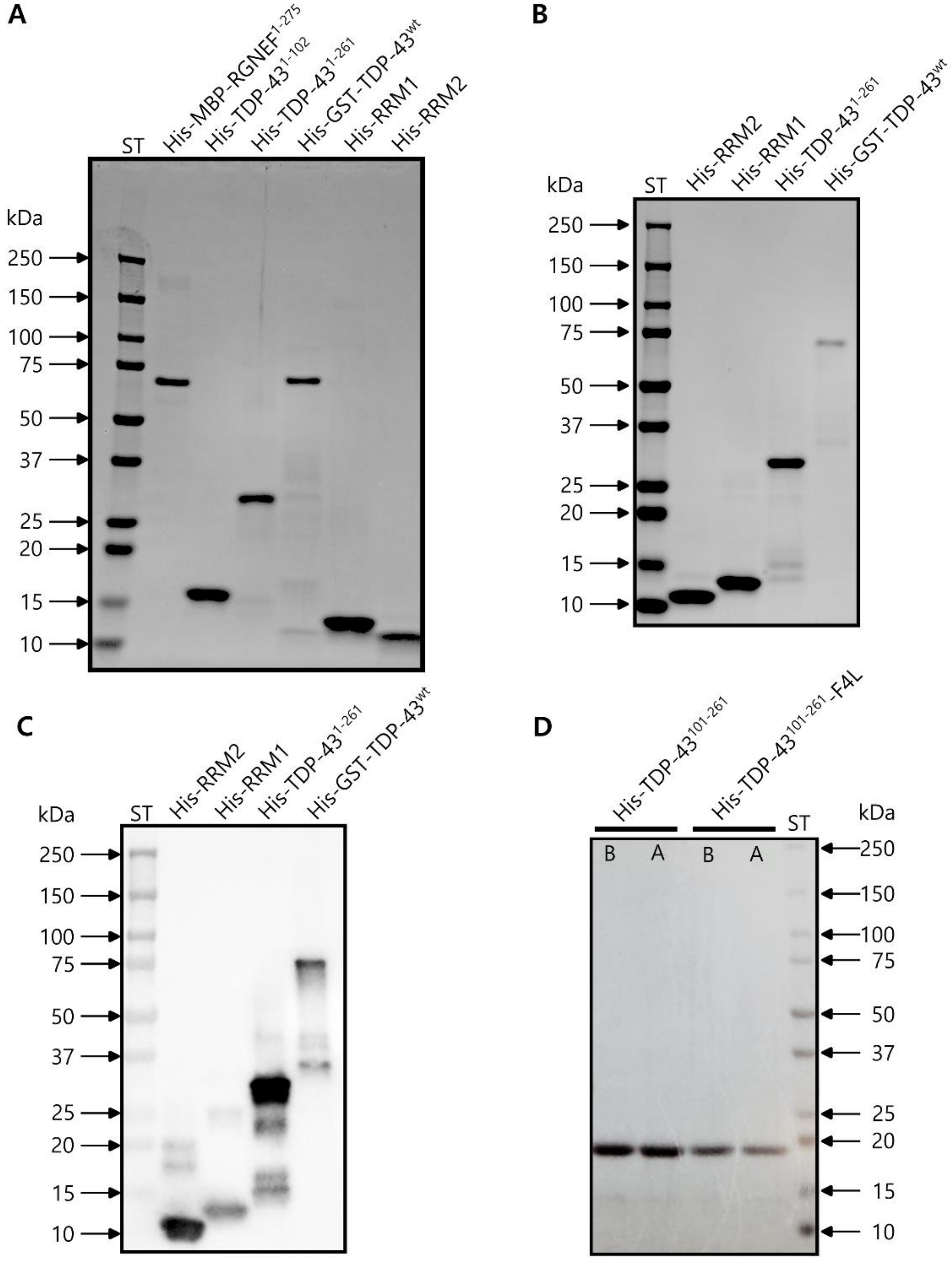

Supplementary Fig. 2 | Gel electrophoresis of recombinant proteins purified for SPR. **A**, 1 μg of purified His-MBP-RGNEF$^{1\text{-}275}$, His-TDP-43$^{1\text{-}102}$, His-TDP-43$^{1\text{-}269}$, His-GST-TDP-43^{wt}, His-RRM-1, and His-RRM-2 were loaded in the gel. **B**, 0.6 μg of purified His-TDP-43$^{1\text{-}269}$, His-RRM-1, and His-RRM-2 samples, and 0.14 μg of purified His-GST-TDP-43^{wt} samples were loaded in the gel. **C**, The same samples (40% less protein) from (b) were also loaded onto a separate precast gradient gel and then transferred to nitrocellulose membrane for western blot analysis using a TDP-43 antibody. The molecular weight standard (ST) was imaged using the colorimetric channel and merged with the chemiluminescent image. **D**, 1.8 μg of purified His-TDP-43$^{101\text{-}261}$ and 0.9 μg His-TDP-43$^{101\text{-}269}$-F4L samples were loaded in the gel (B=before dialysis; A= after dialysis). His: 6x histidine tag; GST: Glutathione S-transferase.

Supplementary Figure 3

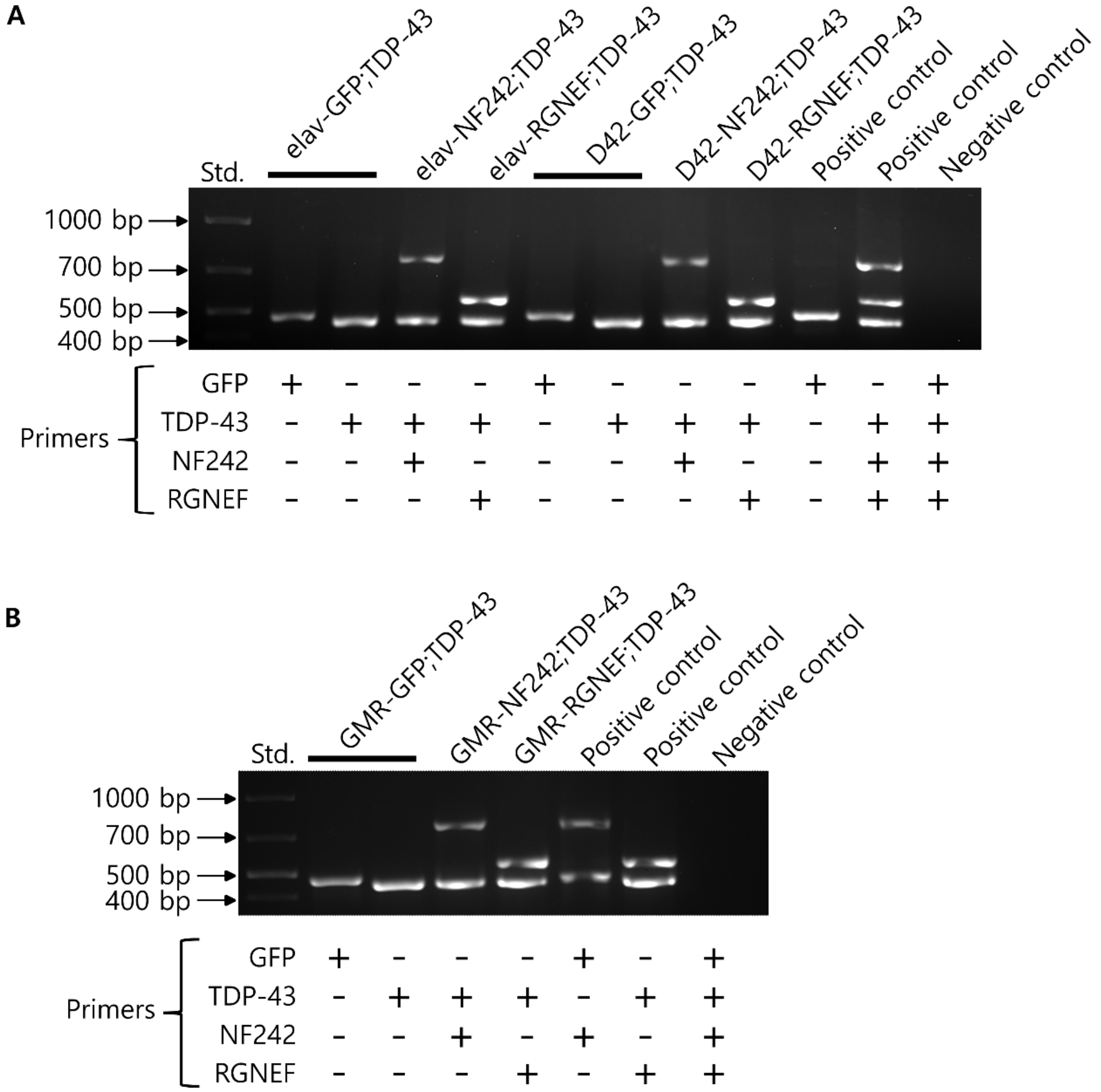

Supplementary Fig. 3 | Expression of genes of interests in transgenic drosophila. A, Expression of GFP, TDP-43, NF242 and RGNEF in double transgenic flies under the elav and D42 drivers. **B**, Expression of GFP, TDP-43, NF242 and RGNEF in double transgenic flies under the GMR driver. As positive controls were used plasmids containing the genes of interest.

Supplementary Figure 4

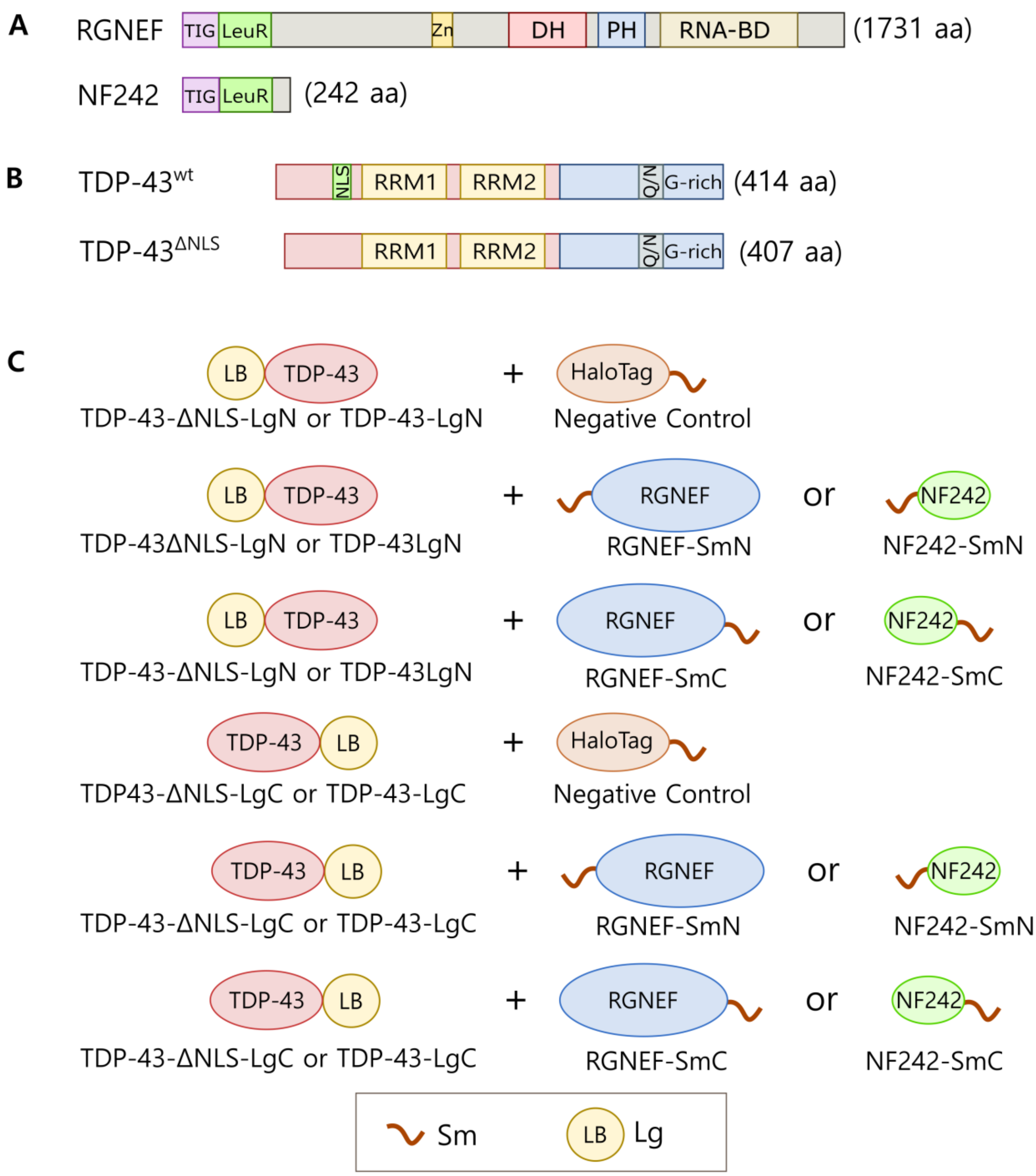

Supplementary Fig. 4 | Constructs used for NanoBiT experiments. A, Schematics of RGNEF and NF242 sequences used in this study. TIG: IPT/TIG domain; LeuR: Leucine-rich domain; Zn: cysteine-rich Zinc binding domain; DH: Dbl homology domain; PH: Pleckstrin homology domain; RNA-BD: RNA-binding domain. **B**, Schematic of the TDP-43^{wt} and TDP-43$^{\Delta NLS}$ sequences used in this study. NLS: Nuclear localization signal; RRM1: RNA recognition motif 1; RRM2: RNA recognition motif 2; Q/N: Glutamine/Asparagine-rich segment; G-rich: Glycine rich region. **C**, Schematic of the NanoBiT constructs used in this study. Sm: Small subunit of luciferase (11 amino acids); Lg: Large subunit of luciferase (17.6 kDa); N: Amino-terminal; C: Carboxy-terminal.

Supplementary Figure 5

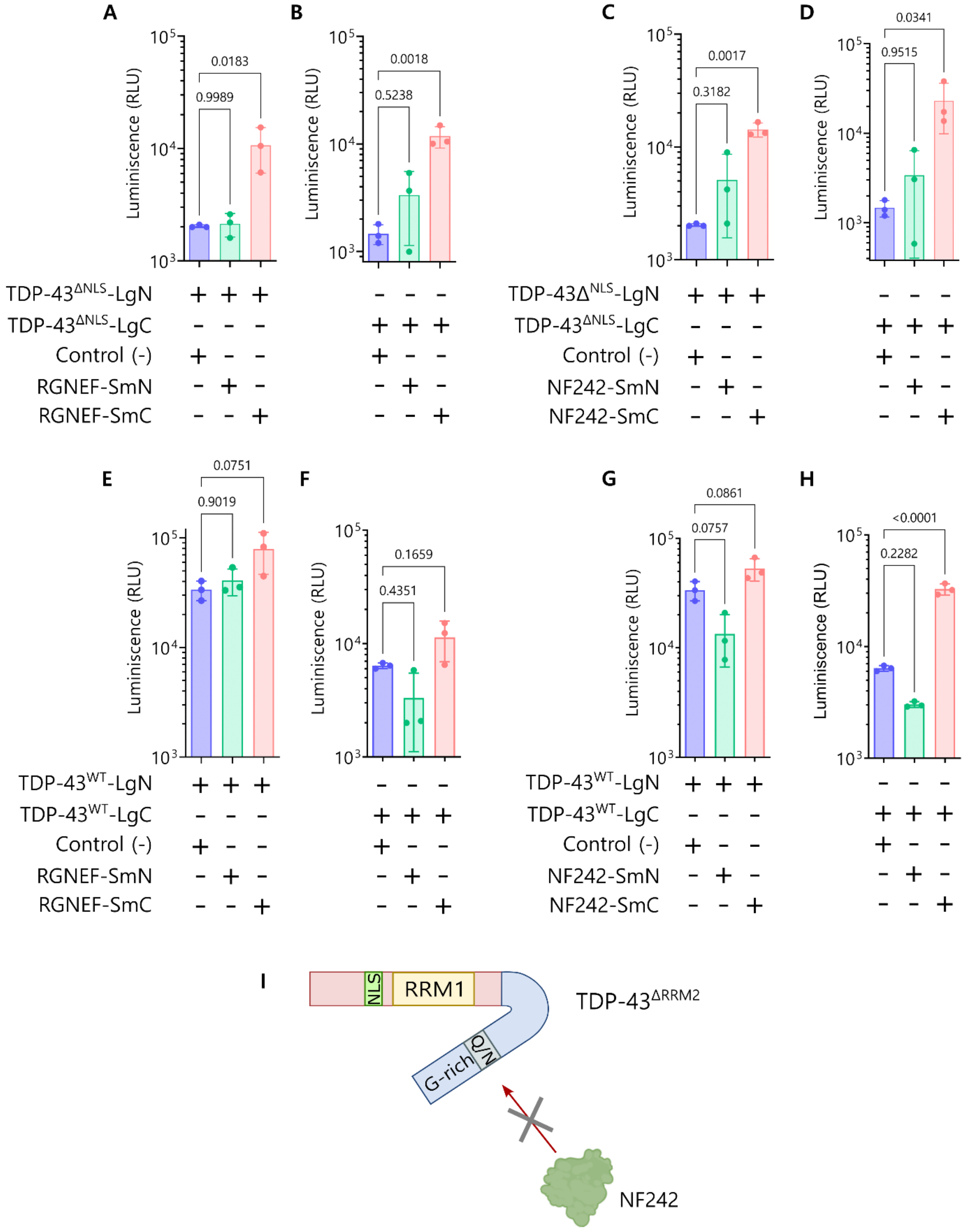

Supplementary Fig. 5 | Interaction between RGNEF or NF242 with TDP-43 using complementation reporter assay (NanoBiT). **A**, Interaction was observed between TDP-43$^{\Delta NLS}$ fused with the large subunit of luciferase (Lg) in the amino-terminal end (TDP-43$^{\Delta NLS}$-LgN) and RGNEF fused with small subunit of luciferase (Sm) in the carboxy-terminal end (RGNEF-SmC; p=0.0183) but not in the amino-terminal end (RGNEF-SmN; p=0.9989). **B**, Interaction was observed between TDP-43$^{\Delta NLS}$ fused with Lg in the carboxy-terminal end (TDP-43$^{\Delta NLS}$-LgC) and RGNEF fused with Sm in the carboxy-terminal end (RGNEF-SmC; p=0.0018) but not in the amino-terminal end (RGNEF-SmN; p=0.5238). **C**, Interaction was observed between TDP-43$^{\Delta NLS}$ fused with Lg in the amino-terminal end (TDP-43$^{\Delta NLS}$-LgN) and NF242 fused with Sm in the carboxy-terminal end (NF242-SmC; p=0.0017) but not in the amino-terminal end (NF242-SmN; p=0.3182). **D**, Interaction was observed between TDP-43$^{\Delta NLS}$ fused with Lg in the carboxy-terminal end (TDP-43$^{\Delta NLS}$-LgC) and NF242 fused with Sm in the carboxy-terminal end (NF242-SmC; p=0.0341) but not in the amino-terminal end (NF242-SmN; p=0.9515). **E**, No interaction was observed between TDP-43^{wt} fused with Lg in the amino-terminal end (TDP-43^{wt}-LgN) and RGNEF fused with Sm in the carboxy-terminal end (RGNEF-SmC; p=0.0751) or in the amino-terminal end (RGNEF-SmN; p=0.9019). **F**, No interaction was observed between TDP-43^{wt} fused with Lg in the carboxy-terminal end (TDP-43^{wt}-LgC) and RGNEF fused with Sm in the carboxy-terminal end (RGNEF-SmC; p=0.1659) or in the amino-terminal end (RGNEF-SmN; p=0.4351). **G**, No interaction was observed between TDP-43^{wt} fused with Lg in the amino-terminal end (TDP-43^{wt}-LgN) and NF242 fused with Sm in the carboxy-terminal end (NF242-SmC; p=0.0861) or in the amino-terminal end (NF242-SmN; p=0.0757). **H**, Interaction was observed between TDP-43^{wt} fused with Lg in the carboxy-terminal end (TDP-43^{wt}-LgC) and NF242 fused with Sm in the carboxy-terminal end (NF242-SmC; p<0.0001) but not in the amino-terminal end (NF242-SmN; p=0.2282). N=3 and 6 technical replicates for each experiment. These results demonstrated that NF242 and TDP-43 fused in the carboxy-terminal end to the luciferase fragments are the best pair for interaction experiments. This configuration was used for the experiments presented in Fig.1. **I**, Schematic proposing an explanation for the lack of interaction between NF242 and TDP-43$^{\Delta RRM2}$ observed in the NanoBiT assay. Our results suggest that the carboxy-terminal region of TDP-43 (in blue) folds over the RRM1 domain blocking the access of NF242.

Supplementary Figure 6

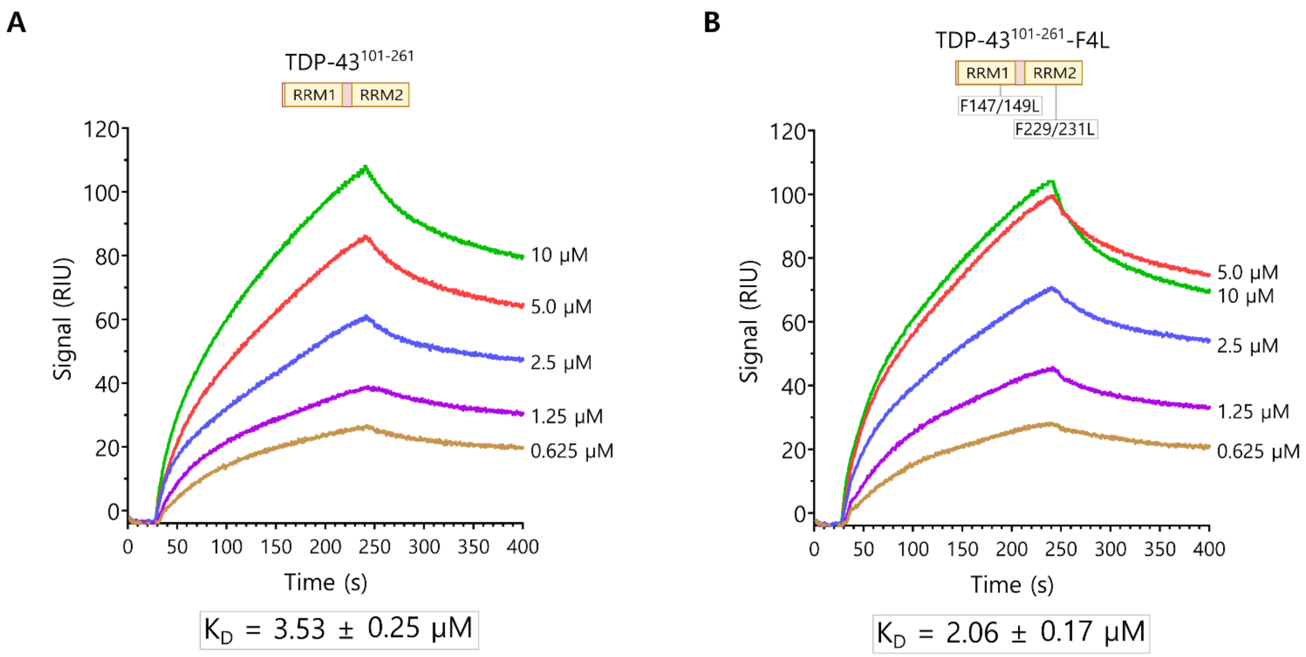

Supplementary Fig. 6 | Interaction between NF242 and TDP-43$^{101\text{-}261}$ or TDP-43$^{101\text{-}261}$-F4L. **A**, Representative SPR sensorgrams showing the interaction between His-MBP-RGNEF$^{1\text{-}275}$ (ligand) and different concentrations of His-TDP-43$^{101\text{-}261}$ (analyte). K_D = 3.53 ± 0.25 μM (n=2). **B**, Representative SPR sensorgrams showing the interaction between His-MBP-RGNEF$^{1\text{-}275}$ (ligand) and different concentrations of His-TDP-43$^{101\text{-}261}$-F4L (analyte). K_D = 2.06 ± 0.17 μM (n=2).

Supplementary Figure 7

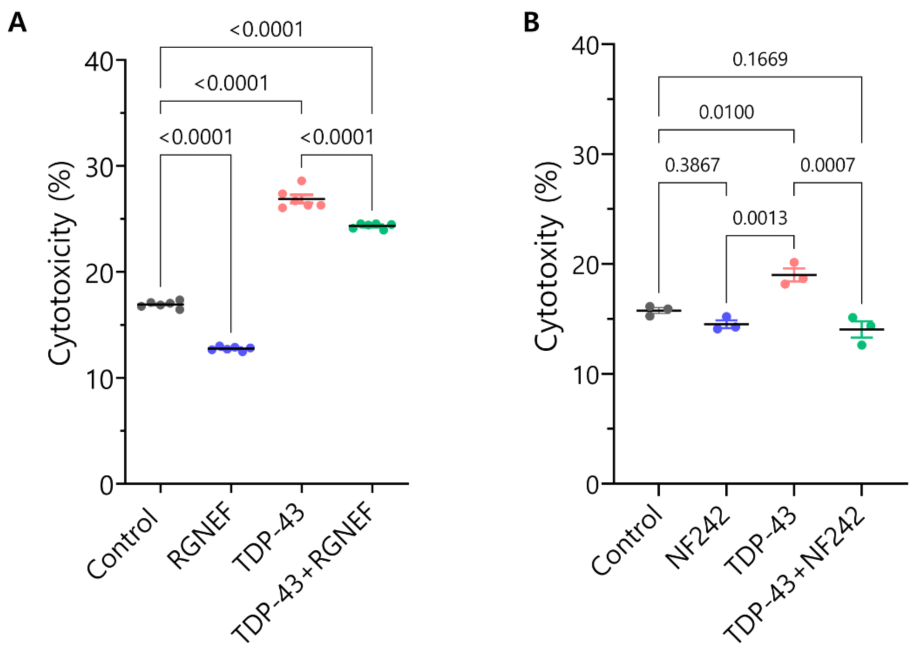

Supplementary Fig. 7 | Cytotoxicity assay of cells transfected with RGNEF, NF242 and TDP-43. a-b, The increase of the cytotoxicity induced by TDP-43 overexpression is reduced with the co-expression of RGNEF (n=6) (**a**) or NF242 (n=3) (**b**). Controls cells transfected with empty vector (control) or vector expressing RGNEF or NF242 alone. RGNEF alone reduced the cytotoxicity in consistence with our previous reports.

Supplementary Figure 8

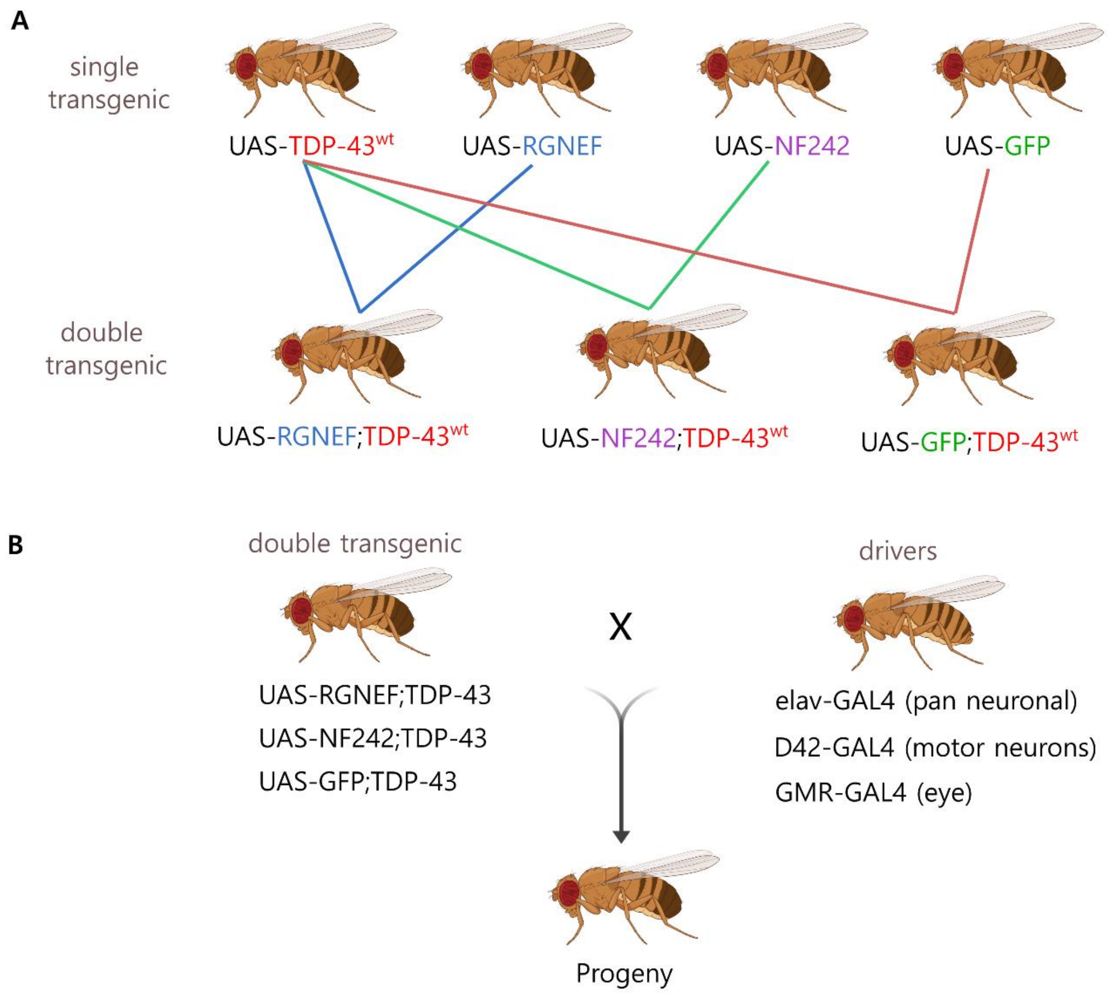

Supplementary Fig. 8 | Transgenic flies used in this study. a, *UAS-TDP-43*, *UAS-RGNEF*, and *UAS-NF242* single transgenic flies were generated for this study. The *UAS-GFP* fly was obtained from Bloomington Drosophila Stock Center. Flies were crossed to generate double transgenic effector lines: *UAS-RGNEF;TDP-43^{wt}*, *UAS-NF242;TDP-43^{wt}*, and *UAS-GFP;TDP-43wt*. **b**, Schematic showing the crosses between effector and driver lines performed in this study.

Supplementary Figure 9

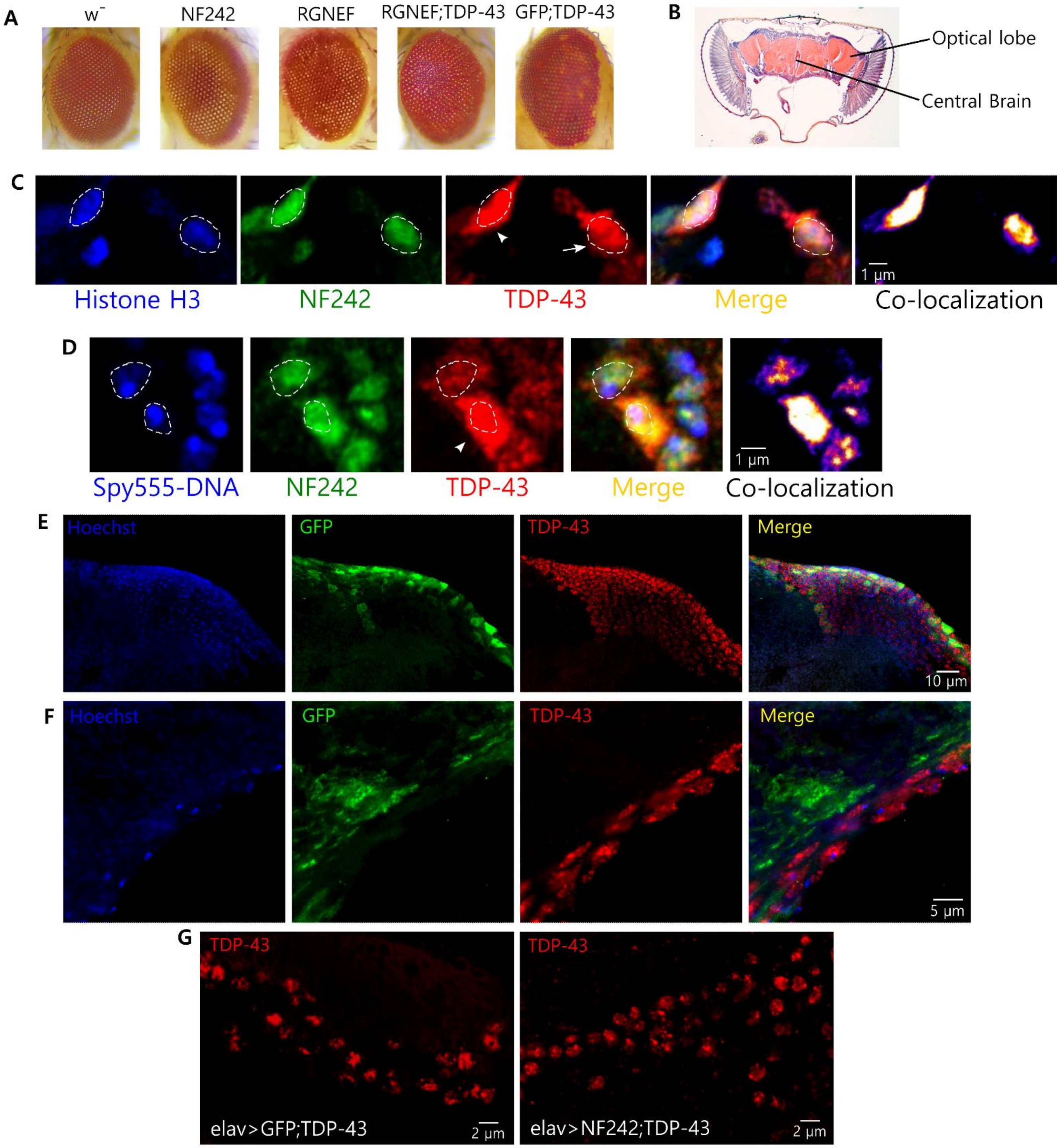

Supplementary Fig. 9 | Immunofluorescence of RGNEF or NF242 with TDP-43 in fruit flies (additional data). **A**, Representative images showing the eye phenotype of lines *GMR>w*$^{-}$ (negative control), *GMR>NF242*; *GMR>RGNEF*, *GMR>RGNEF;TDP-43*^{wt}, and *GMR>GFP;TDP-43*^{wt}. Both *GMR>RGNEF*, *GMR>RGNEF;TDP-43*^{wt}, show a distinctive eye phenotype that is different from the pathological *GMR>GFP;TDP-43*^{wt}. The *GMR>w*$^{-}$ and *GMR>NF242* lines show a normal eye phenotype. **B**, Hematoxylin-eosin staining showing an adult *Drosophila* head. The brain regions, including the optical lobes, analyzed in this study are indicated. **C-D,** Confocal images at higher magnification of adult elav>NF242;TDP-43^{wt} fly tissue showing the co-aggregation between NF242 and TDP-43wt in brain cells. Nuclei marked using anti-Histone H3 antibody (**C**) or SPY555-DNA (**D**) are indicated with dashed lines. Arrow shows nuclear co-localization and arrowheads cytoplasmic co-localization. **E**, Immunofluorescence of adult *elav>GFP;TDP-43*^{wt} fly brain tissue showing the localization GFP and TDP-43 in neurons. **F**, Higher magnification of adult *elav>GFP;TDP-43*^{wt} fly brain tissue showing the aggregation of TDP-43 in neurons. **G**, Immnuofluorescence showing TDP-43 pathology in the brain of *elav>GFP;TDP-43*^{wt} and *elav>NF242;TDP-43*^{wt} flies. No difference in the TDP-43 pathology is observed between either fly.

Supplementary Figure 10

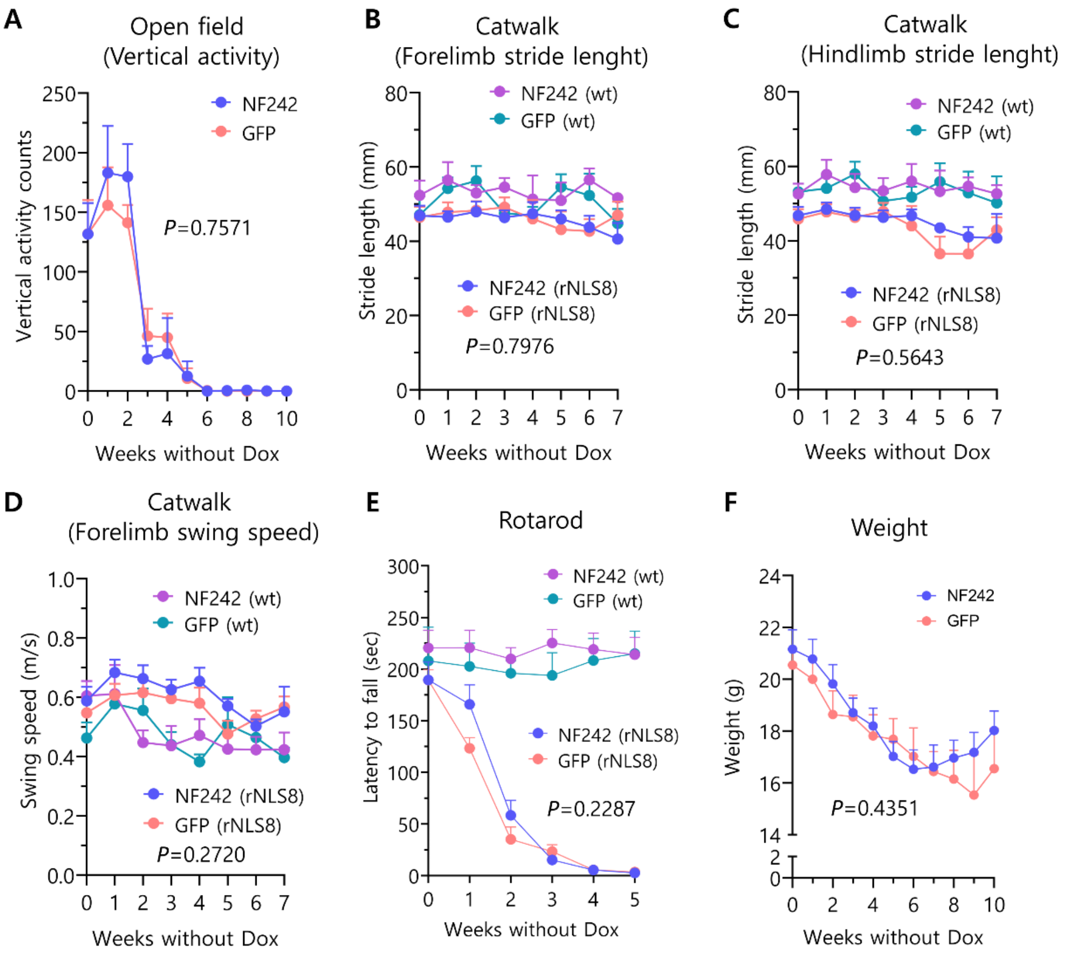

Supplementary Fig. 10| Ectopic expression of NF242 in rNLS8 mice (Additional data). A, Open field test comparing rNLS8 mice injected with AAV9/GFP (n=12) or AAV9/NF242 (n=12) shows no difference in vertical activity between the two groups (p=0.7571). **B-D**, Catwalk quantification comparing rNLS8 and wt mice injected with AAV9/GFP or AAV9/NF242 (n=12 for each group of rNLS8 mice; n=6 for each group of wt mice). The two rNLS8 groups show no difference in forelimb stride length (p=0.7976) (**B**), hindlimb stride length (p=0.5643) (**C**), and forelimb swing speed (p=0.2720) (**D**). **E**, Rotarod test show no difference between rNLS8 mice injected with AAV9/GFP (n=12) or AAV9/NF242 (n=12) (p=0.2741). Wt mice injected with AAV9/GFP (n=6) or AAV9/NF242 (n=6) are shown as healthy controls. **F**, Weight of rNLS8 mice injected with AAV9/GFP (n=10) or AAV9/NF242 (n=9). No significant difference was found between the groups (p=0.4351).

Supplementary Figure 11

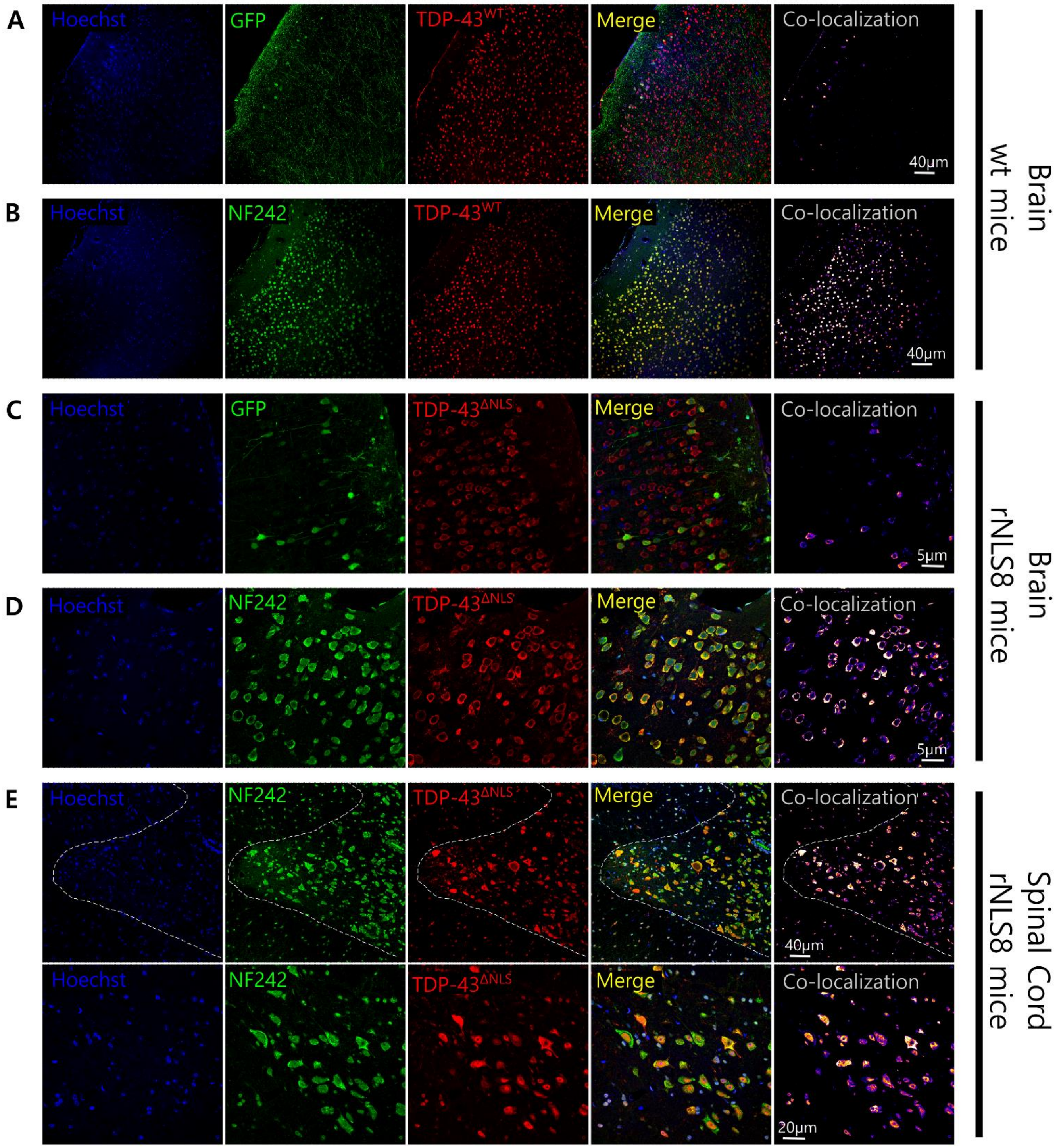

Supplementary Fig. 11 | Pathology of rNLS8 mice expressing ectopic NF242 (Additional data). A, Expression of GFP and endogenous TDP-43^{wt} in the brain cortex (cortical layer II-III) of wild-type mouse injected with AAV9/GFP, after 3 weeks without Dox. **B**, Expression of NF242 and endogenous TDP-43^{wt} in the brain cortex (cortical layer II-III) of wt mouse injected with AAV9/NF242, after 3 weeks without Dox. **C**, Expression of GFP and TDP-43$^{\Delta NLS}$ in the brain cortex (cortical layer II-III) of rNLS8 mouse injected with AAV9/GFP, after 3 weeks without Dox. **D**, Co-localization of NF242 with TDP-43$^{\Delta NLS}$ in the brain cortex (cortical layer II-III) of rNLS8 mouse injected with AAV9/NF242, after 3 weeks without Dox. **e**, Two magnifications showing the co-localization of NF242 with TDP-43$^{\Delta NLS}$ in the spinal cord of rNLS8 mouse injected with AAV9/NF242, after 3 weeks without Dox. The gray matter is separated from the white matter by a dashed white line.

Supplementary Figure 12

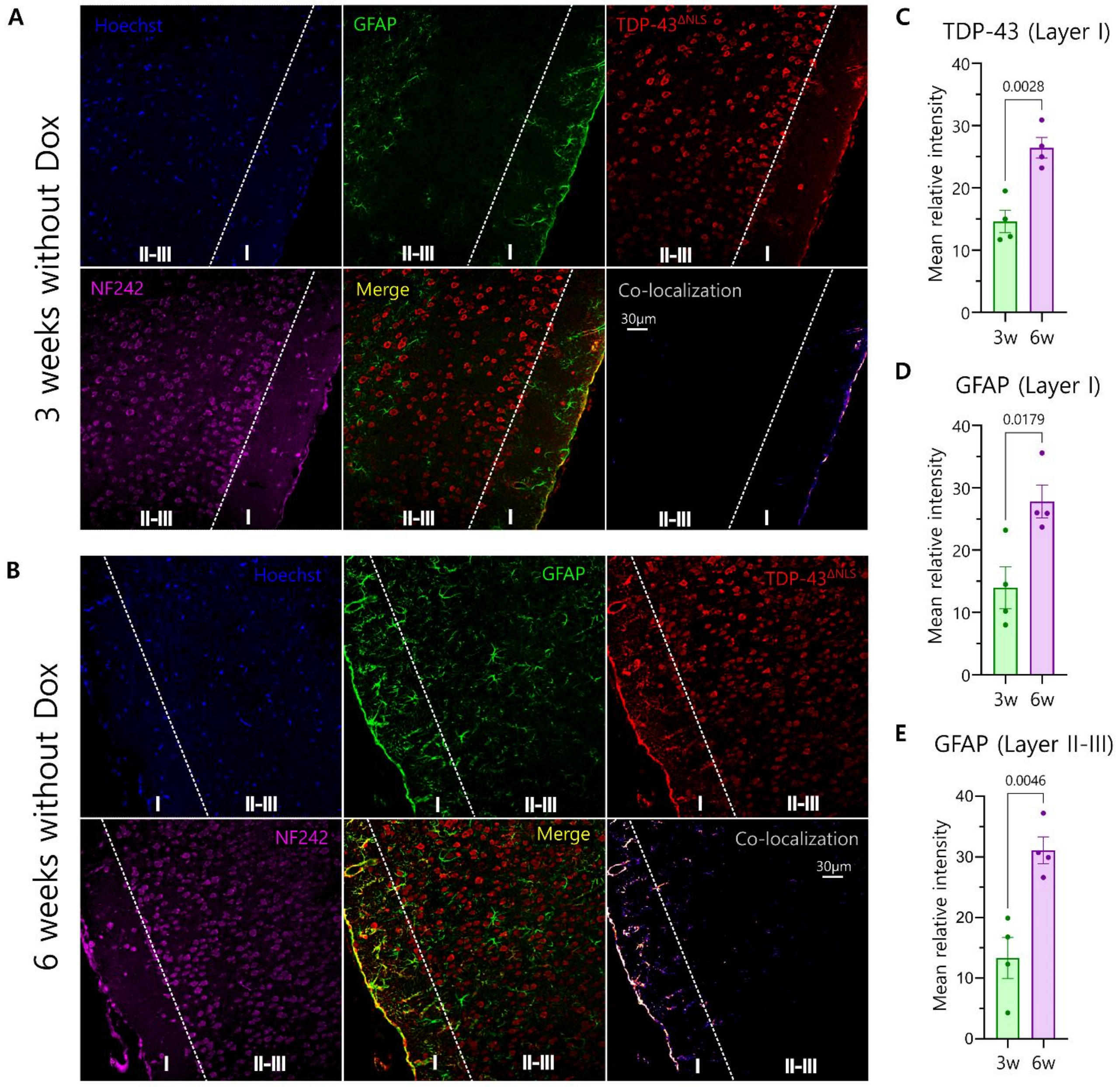

Supplementary Fig. 12 | Comparison of rNLS8 mice brain expressing ectopic NF242 between week 3 and 6. **A,** Localization analysis of GFAP and TDP-43ΔNLS in the cortex of rNLS8 mouse injected with AAV9/NF242, after 3 weeks without Dox. Notice the low levels of TDP-$43^{\Delta NLS}$ in the cortical layer I of the brain and the absence of co-localization between TDP-43ΔNLS and GFAP in that region. **B**, Localization analysis of GFAP and TDP-$43^{\Delta NLS}$ in the cortex of rNLS8 mouse injected with AAV9/NF242, after 6 weeks without Dox. Notice the spreading of TDP-43 pathology in the cortical layer I of the brain and the intensive co-localization between GFAP and TDP-$43^{\Delta NLS}$ in that region. **C**, Quantification of the increase of TDP-$43^{\Delta NLS}$ amount in the cortical layer I of the brain from 3 to 6 weeks without Dox (n=4, p=0.0028). **D**, Quantification of the increase of GFAP amount in the cortical layer I of the brain from 3 to 6 weeks without Dox (n=4, p=0.0179). **E**, Quantification of the increase of GFAP amount in the cortical layer II-III of the brain from 3 to 6 weeks without Dox (n=4, p=0.0046). The cortical layer I and II-III are indicated in all images.

Supplementary Table 1

Reagent or Resource	Source	Catalog number
Antibodies		
Anti-flag (goat)	Novus Biologicals	NB600-344
Anti-GFP (rabbit)	Abcam	ab6673
Anti-TDP-43 (rabbit)	Proteintech	10782-2-AP
Anti-TDP-43 (mouse)	Abcam	ab104223
Anti-Iba1 (rabbit)	Wako	019-197-41
Anti-GFAP (mouse)	BD	556330
Anti-Histone H3 (rabbit)	Abcam	ab1791
Anti-rabbit HRP conjugated (swine)	Dako	P0399
Anti-goat Alexa 488 (donkey)	Invitrogen	A11055
Anti-rabbit Alexa 555 (donkey)	Invitrogen	A31572
Anti-mouse Alexa 555 (donkey)	Invitrogen	A31570
Anti-rabbit Alexa 594 (donkey)	Invitrogen	A21207
Anti-rabbit Alexa 680 (donkey)	Invitrogen	A10043
Chemicals and kits		
Hoechst 32258	Sigma	B-1155
SPY555-DNA	Spirochrome	SC201
DMEM	Gibco - Life technologies	11995-065
Fetal Bovine Serum	Gibco - Life technologies	12483-020
Pen Strep (Penicillin Streptomycin)	Gibco - Life technologies	15140-122
Plasmocin	InvivoGen	ant-mpt
Attachment Factor 1X	Gibco - Life technologies	S-006-100
Lipofectamine 2000	ThermoFisher Scientific	11668019
Magnetofectamine O2	OZ Biosciences	MTX2-0750
Phusion High-Fidelity DNA Polymerase	ThermoFisher Scientific	F530L
Western Lightning Plus ECL	PerkinElmer	NEL10400EA
Halt™ Protease Inhibitor Cocktail	ThermoFisher Scientific	78425
NEBExpress® Ni Spin Columns	New England Biolabs	S1427S
Slide-A-Lyzer™ MINI Dialysis Devices	ThermoFisher Scientific	69570
Pierce™ BCA Protein Assay Kit	ThermoFisher Scientific	23227
Recombinant *E. coli* MBP His Protein	Novus Biologicals	NBP2-22654
CytoTox-Glo™ Cytotoxicity Assay	Promega	G9291
NanoBit Protein:Protein Interaction System	Promega	N2014
Nano-Glo Live Cell Assay System	Promega	N2012
Recombinant DNA		
Plasmid: SmBiT-PRKACA Control Vector	Promega	N2014
Plasmid: LgBiT-PRKAR2A Control Vector	Promega	N2014
Plasmid: NanoBiT Negative Control Vector	Promega	N2014

Plasmid: pBiT-N-Lg-TDP-43^{wt}	This paper	N/A
Plasmid: pBiT-C-Lg-TDP-43^{wt}	This paper	N/A
Plasmid: pBiT-N-Lg-TDP-43$^{\Delta NLS}$	This paper	N/A
Plasmid: pBiT-C-Lg-TDP-43$^{\Delta NLS}$	This paper	N/A
Plasmid: pBiT-C-Lg-TDP-43$^{(1\text{-}366)}$	This paper	N/A
Plasmid: pBiT-C-Lg-TDP-43$^{(1\text{-}274)}$	This paper	N/A
Plasmid: pBiT-C-Lg-TDP-43$^{\Delta RRM\text{-}1\text{-}2}$	This paper	N/A
Plasmid: pBiT-C-Lg-TDP-43$^{\Delta RRM\text{-}1}$	This paper	N/A
Plasmid: pBiT-C-Lg-TDP-43$^{\Delta RRM\text{-}2}$	This paper	N/A
Plasmid: pBiT-C-Lg-TDP-43$^{(1\text{-}192)}$	This paper	N/A
Plasmid: pBiT-N-Sm-RGNEF	This paper	N/A
Plasmid: pBiT-C-Sm-RGNEF	This paper	N/A
Plasmid: pBiT-N-Sm-NF242	This paper	N/A
Plasmid: pBiT-C-Sm-NF242	This paper	N/A
Plasmid: pBiT-C-Sm-NF242$^{mut77\text{-}79}$	This paper	N/A
Plasmid: pBiT-C-Sm-NF242$^{\Delta 77\text{-}79\ P81G}$	This paper	N/A
Plasmid: pQE30-TDP-43$^{1\text{-}269}$	Emanuele Buratti (Italy)	N/A
Plasmid: pQE30-TDP-43$^{101\text{-}261}$	Emanuele Buratti (Italy)	N/A
Plasmid : pBAD-HisA-GST-TDP-43Cri	Emanuele Buratti (Italy)	N/A
Plasmid: pQE30-TDP-43-RRM1	This paper	N/A
Plasmid: pQE30-TDP-43-RRM2	This paper	N/A
Plasmid: pDEST566-RGNEF-275	Murray Junop (Canada)	N/A

Supplementary Table 2

Insert	Backbone	Vector name	Protein product
TDP-43	pBiT1.1-N [TK LgBiT]	pBiT-N-Lg-TDP-43	Lg-TDP-43
	pBiT1.1-C [TK LgBiT]	pBiT-C-Lg-TDP-43	TDP-43-Lg
TDP-43-ΔNLS	pBiT1.1-N [TK LgBiT]	pBiT-N-Lg-TDP-43-ΔNLS	Lg-TDP-43-ΔNLS
	pBiT1.1-C [TK LgBiT]	pBiT-C-Lg-TDP-43-ΔNLS	TDP-43-ΔNLS-Lg
RGNEF	pBiT2.1-N [TK SmBiT]	pBiT-N-Sm-RGNEF	Sm-RGNEF
	pBiT2.1-C [TK SmBiT]	pBiT-C-Sm-RGNEF	RGNEF-Sm
NF242	pBiT2.1-N [TK SmBiT]	pBiT- N-Sm-NF242	Sm-NF242
	pBiT2.1-C [TK SmBiT]	pBiT-C-Sm-NF242	NF242-Sm

Supplementary Table 3

(Fly lines from stock centers)

Name	Stock#	Company	Chr.	Expression
GMR	1104	Bloomington	2	Eye*
D42	8816	Bloomington	3	Motor Neuron
elav	458	Bloomington	1	Pan-Neuronal†
w-	60000	Vienna Drosophila Resource Center	1	---
GFP; Dr/Sb	60292	Bloomington	2	---
C9-36R	58688	Bloomington	2	---

* = Provides strong expression in all cells behind the morphogenetic furrow.

† = Begins expression at stage 12 of embryonic development.

Supplementary Table 4

Gene expressed	Driver	Genotype	Name in this manuscript
RGNEF	elav	$\frac{w[1118]}{elav-GAL4};\frac{UAS-RGNEF.myc,mw+}{+};\frac{+}{+}$	*elav>RGNEF*
NF242	elav	$\frac{w[1118]}{elav-GAL4};\frac{UAS-flag.NF242,mw+}{+};\frac{+}{+}$	*elav>NF242*
	GMR	$\frac{w[1118]}{+};\frac{UAS-flag.NF242,mw+}{GMR-GAL4};\frac{+}{+}$	*GMR>NF242*
GFP + TDP-43	GMR	$\frac{w[1118]}{+};\frac{UAS-2xEGFP}{GMR-GAL4};\frac{UAS-hTDP43,mw+}{+}$	*GMR>GFP;TDP-43^{wt}*
	elav	$\frac{w[1118]}{elav-GAL4};\frac{UAS-2xEGFP}{+};\frac{UAS-hTDP43,mw+}{+}$	*elav>GFP;TDP-43^{wt}*
	D42	$\frac{w[1118]}{+};\frac{UAS-2xEGFP}{+};\frac{UAS-hTDP43,mw+}{D42-GAL4}$	*D42>GFP;TDP-43^{wt}*
RGNEF + TDP-43	GMR	$\frac{w[1118]}{+};\frac{UAS-RGNEF.myc,mw+}{GMR-GAL4};\frac{UAS-hTDP43,mw+}{+}$	*GMR>RGNEF;TDP-43^{wt}*
	elav	$\frac{w[1118]}{elav-GAL4};\frac{UAS-RGNEF.myc,mw+}{+};\frac{UAS-hTDP43,mw+}{+}$	*elav>RGNEF;TDP-43^{wt}*
	D42	$\frac{w[1118]}{+};\frac{UAS-RGNEF.myc,mw+}{+};\frac{UAS-hTDP43,mw+}{D42-GAL4}$	*D42>RGNEF;TDP-43^{wt}*
NF242 + TDP-43	GMR	$\frac{w[1118]}{+};\frac{UAS-flag.NF242,mw+}{GMR-GAL4};\frac{UAS-hTDP43,mw+}{+}$	*GMR>NF242;TDP-43^{wt}*
	elav	$\frac{w[1118]}{elav-GAL4};\frac{UAS-flag.NF242,mw+}{+};\frac{UAS-hTDP43,mw+}{+}$	*elav>NF242;TDP-43^{wt}*
	D42	$\frac{w[1118]}{+};\frac{UAS-flag.NF242,mw+}{+};\frac{UAS-hTDP43,mw+}{D42-GAL4}$	*D42>NF242;TDP-43^{wt}*

Supplementary Table 5

Primer name	Sequence	Amplicon size
TDP-43-F	5’ GGACTTGATCATTAAAGGAATCAGCGTTC 3’	447 bp
TDP-43-R	5’ CTGCCCGACCCTGCATTGGATG 3’	
RGNEF-F	5’ GCCCCGAGGTAATGGAACTTAATCG 3’	550 bp
RGNEF-R	5’ TAAACAATATTTTCTTTGGCTCCATCTCCAGT 3’	
NF242-F	5’ ATGACAAGATGGAGTTGAGCTGCAGCGAAG 3’	734 bp
NF42-R	5’ GTAATGCAAGGAGGCTTCTTCACTG 3’	
GFP-F	5’ CCACCCTCGTGACCACCCTGA 3’	474 bp
GFP-R	5’ CGCGCTTCTCGTTGGGGTCTT 3’	
18S-F	5’ AGTATGGTTGCAAAGCTGAAACTTAAAGGA 3’	222bp
18S-R	5’ GAGTCTCGTTCGTTATCGGAATTAACC 3’	
qNano Lg-F	5´- GAACAGACAGCCGCCTACAACCTG-3´	428bp
qNano Lg-R	5´- ACTCGGAACAGCATGGAGCCGTC-3´	
qNano Sm-F	5´-GGTGACCGGCTACCGGCTG-3´	375bp
qNano RGNEF/NF242 N-R	5´- GCCAGCCTGCAAGCCATGTTGTC-3’	
qNano RGNEF C-F	5´- GTCAGTCACAAACTGTGGACAGCCG-3´	397 bp
qNano Sm C -R	5´- CGAACAGCCGGTAGCCGGTC-3’	
qNano NF242 C-F	5´- GCACTGCCTGCCTTGGATGAGG-3´	412 bp
qNano Sm C -R	5´- CGAACAGCCGGTAGCCGGTC-3’	

Supplementary Table 6

(Antibody dilutions)

**Antibody**	**Dilution**
Anti-flag (goat)	1/200
Anti-GFP (rabbit)	1/400
Anti-TDP-43 (rabbit)	1/250 (IF) 1/4000 (WB)
Anti-TDP-43 (mouse)	1/250
Anti-Iba1 (rabbit)	1/100
Anti-GFAP (mouse)	1/100
Anti-goat Alexa 488 (donkey)	1/1,000
Anti-rabbit Alexa 555 (donkey)	1/1,000
Anti-mouse Alexa 555 (donkey)	1/1,000
Anti-rabbit Alexa 594 (donkey)	1/1,000
Anti-rabbit Alexa 680 (donkey)	1/1,000

Supplementary Methods

Protein Purification

For purification of His-GST-TDP-43^{wt}, transformed *E. coli* BL21 (DE3) cells were grown in LB medium. The expression was induced by adding 0.2% L-Arabinose and the culture was grown for an additional 4 hours at 37 ºC. The cells were harvested by centrifugation and resuspended in lysis buffer (20 mM Tris-HCl pH 7.5, 500 mM NaCl, 20 mM Imidazole, 10% Glycerol, 1 mM PMSF, and 1x Protease Inhibitor Cocktail). The cells were lysed using EmulsiFlex-C3 (Avestin) and Bioruptor UCD-200 (Diagenode). The lysate was clarified by centrifugation and the supernatant was loaded onto a column packed with Ni-IDA resin (GE Life Sciences) pre-equilibrated with wash buffer (20 mM Tris-HCl pH 7.5, 500 mM NaCl, 20 mM Imidazole, 10% Glycerol). The column was washed with wash buffer, and the bound protein was eluted with elution buffer (20 mM Tris-HCl, pH 7.5, 500 mM NaCl, 250 mM Imidazole, 10% Glycerol). The eluted protein was passed through a GPC column (Superdex 75 Increase 10/300 GL, GE Life Sciences) equilibrated with buffer (20 mM Tris-HCl pH 7.5, 150 mM NaCl, 10% Glycerol). The major peak obtained in GPC was pooled and further concentrated using Amicon Ultra-15 centrifugal filters (Millipore) with a molecular weight cutoff of 10 kDa. The purified protein was dialyzed to 0.2 µm-filtered SPR running buffer (20 mM Hepes pH 7.4, 50 mM NaCl, 50 mM KCl, 0.5 mM MgCl2, 0.05% Tween-20) using 10 kDa molecular weight cut off Slide-A-Lyzer™ MINI Dialysis Devices following manufacturer instructions immediately prior to SPR analysis.

For the purification of His-TDP-43$^{1-269}$, His-RRM-1, His-RRM-2, and His-TDP-43$^{101-261}$ M15 bacteria were grown in liquid LB containing ampicillin (Amp) and kanamycin (Kan) (ampicillin resistance encoded on the plasmid and kanamycin resistance encoded by the M15 strain of *E. coli*) at 37 °C overnight. For His-MBP-RGNEF$^{1-275}$ bacteria were grown in LB containing only Amp (BL21DE3T1R *E. coli*). The expression was induced with 1 mM isopropyl β-d-1-thiogalactopyranoside (IPTG) at 18°C for 16 hours. Induced cultures were collected, centrifuged, and then resuspended in cold lysis buffer containing 1x PBS, 1x Halt™ Protease Inhibitor Cocktail and 1 mg/ml lysozyme for TDP-43 proteins and 800 mM NaCl, 20 mM Tris-HCl pH 8.0, and 10% v/v glycerol for His-MBP-RGNEF$^{1-275}$. Pellets were incubated on ice for 30 minutes and lysed by

sonication or French Press. Lysates containing His-TDP-43$^{1-269}$, His-RRM-1, and His-RRM-2 were cleared by centrifugation and applied to NEBExpress® Ni Spin Columns following manufacturer directions. For His-MBP-RGNEF$^{1-275}$ Ni-IMAC was used on the ÄKTA Start Protein Purification System with a 5 mL HiTrap HP column (Cytiva Life Sciences, United States). Used buffers were: lysis buffer as described above, wash buffer (1x PBS, 300 mM NaCl, 5 mM imidazole for His-TDP-43$^{1-269}$, His-RRM-1, and His-RRM-2; and an imidazole gradient up to 800 mM NaCl, 300 mM imidazole, 20 mM Tris-HCl pH 8.0, and 10% v/v glycerol for His-MBP-RGNEF$^{1-275}$), and elution buffer (1x PBS, 1 mM DTT, 0.1% v/v Tween-20, 500 mM imidazole for His-TDP-43$^{1-269}$, His-RRM-1, and His-RRM-2; and 800 mM NaCl, 300 mM imidazole, 20 mM Tris-HCl pH 8.0, and 10% v/v glycerol for His-MBP-RGNEF$^{1-275}$). Purified proteins were visualized by SDS-PAGE. Eluted samples used for SPR analysis were incubated on ice and dialyzed to 0.2 µm-filtered SPR running buffer using 10 kDa molecular weight cut-off Slide-A-Lyzer™ MINI Dialysis Devices according to the manufacturer instructions immediately prior to SPR analysis.

Protein concentrations were determined using the BCA microplate assay and BSA standards according to the manufacturer directions.

Supplementary videos

Supplementary video 1. Video showing the phenotype of a rNLS8 mouse injected with AAV9/GFP after 3 weeks without Dox.

Supplementary video 2. Video showing the phenotype of a rNLS8 mouse injected with AAV9/GFP after 5 weeks without Dox.

Supplementary video 3. Video showing the phenotype of a rNLS8 mouse injected with AAV9/NF242 after 3 weeks without Dox.

Supplementary video 4. Video showing the phenotype of a rNLS8 mouse injected with AAV9/NF242 after 5 weeks without Dox.
